# Host MOV10 is induced to restrict herpes simplex virus 1 lytic infection by promoting type I interferon response

**DOI:** 10.1371/journal.ppat.1010301

**Published:** 2022-02-14

**Authors:** Xiyuan Yang, Ze Xiang, Zeyu Sun, Feiyang Ji, Keyi Ren, Dongli Pan

**Affiliations:** 1 State Key Laboratory for Diagnosis and Treatment of Infectious Diseases, The First Affiliated Hospital, Zhejiang University School of Medicine, Hangzhou, Zhejiang, China; 2 Department of Medical Microbiology and Parasitology, Zhejiang University School of Medicine, Hangzhou, Zhejiang, China; Florida State University, UNITED STATES

## Abstract

Moloney leukemia virus 10 protein (MOV10) is an interferon (IFN)-inducible RNA helicase implicated in antiviral activity against RNA viruses, yet its role in herpesvirus infection has not been investigated. After corneal inoculation of mice with herpes simplex virus 1 (HSV-1), we observed strong upregulation of both MOV10 mRNA and protein in acutely infected mouse trigeminal ganglia. MOV10 suppressed HSV-1 replication in both neuronal and non-neuronal cells, and this suppression required the N-terminus, but not C-terminal helicase domain of MOV10. MOV10 repressed expression of the viral gene ICP0 in transfected cells, but suppressed HSV-1 replication independently of ICP0. MOV10 increased expression of type I IFN in HSV-1 infected cells with little effect on IFN downstream signaling. Treating the cells with IFN-α or an inhibitor of the IFN receptor eliminated MOV10 suppression of HSV-1 replication. MOV10 enhanced IFN production stimulated by cytoplasmic RNA rather than DNA. IKKε co-immunoprecipitated with MOV10 and was required for MOV10 restriction of HSV-1 replication. Mass spectrometry identified ICP27 as a viral protein interacting with MOV10. Co-immunoprecipitation results suggested that this interaction depended on the RGG box of ICP27 and both termini of MOV10. Overexpressed ICP27, but not its RGG-Box deletion mutant, rendered MOV10 unable to regulate HSV-1 replication and type I IFN production. In summary, MOV10 is induced to restrict HSV-1 lytic infection by promoting the type I IFN response through an IKKε-mediated RNA sensing pathway, and its activity is potentially antagonized by ICP27 in an RGG box dependent manner.

## Author summary

Herpes simplex virus 1 (HSV-1) is a ubiquitous DNA virus that can cause various human diseases. Upon HSV-1 invasion, the host elicited the type I interferon (IFN) response as the first line of defense, in which numerous host factors are induced to restrict viral infection, yet our knowledge about these restriction factors remains limited. Here we show that during HSV-1 acute infection Moloney leukemia virus 10 protein (MOV10) was induced to restrict HSV-1 productive infection. MOV10 restricted HSV-1 replication by promoting type I IFN production through an IKKε-mediated RNA sensing pathway. Moreover, we identified ICP27 as a viral protein that can interact with MOV10 and antagonize its antiviral activity. Thus we establish MOV10 as a host restriction factor against a herpesvirus for the first time and expand our knowledge about how viral and host proteins modulate the IFN response.

## Introduction

Herpes simplex virus 1 (HSV-1) is a double-stranded DNA virus encoding more than 80 proteins. Upon invasion of a host, HSV-1 initially undergoes productive (lytic) infection in peripheral tissues characterized by sequential expression of immediate-early, early and late genes, and abundant production of viral particles [1]. After lytic infection, HSV-1 establishes latent infection in sensory neurons, where the viral genome is permanently present in the neuronal nucleus in an epigenetically silenced state with the only gene products abundantly produced being the latency-associated transcripts and some microRNAs (miRNAs). Upon stimulation, this state is reversed during a reactivation process that allows the virus to re-enter the lytic state and spread further.

The type I interferon (IFN) response provides the first line of host defense against HSV-1 invasion [1]. Recognition of pathogen associated molecular patterns, such as cytoplasmic DNA and RNA, by cellular pattern recognition receptors triggers signal transduction cascades leading to stimulation of type I IFN production. The upregulated IFN then signals the IFN receptors on the plasma membrane to activate the downstream Janus kinases (Jak)/signal transducers and activators of transcription (STAT) pathway, thereby inducing the expression of hundreds of interferon-stimulated genes (ISGs), which collectively establishes an antiviral state [2]. Type I IFN is not only crucial for HSV-1 clearance from non-neuronal cells, but its neuronal signaling also protects the host from HSV-1 replication and pathogenesis [3–5]. To overcome the antiviral state, HSV-1 has involved various strategies that evade or subvert the IFN response [6,7].

Many host RNA helicases play a role in innate immunity against viruses [8]. Retinoic-acid-inducible protein I (RIG-I) and melanoma-differentiation-associated gene 5 (MDA5) are the main cytosolic sensors of viral double-stranded RNA [9,10]. They recruit mitochondrial antiviral signaling protein (MAVS) to activate the TANK binding kinase (TBK1)/IκB kinase ε (IKKε) complex, which phosphorylates and activates IFN regulatory factor 3 (IRF3) or IRF7 before these IRFs enter the nucleus to stimulate type I IFN transcription [11,12]. This cytoplasmic RNA sensing pathway mediates the IFN response to both RNA and DNA viruses [13]. Some other RNA helicases, such as DDX3, DDX41, DDX1 and LGP2, are also involved in antiviral innate immunity [14–17]. Conversely, some viral proteins antagonize these RNA helicases. For example, HSV-1 UL37 deamidates RIG-I to block RNA sensing [18,19] and HSV-1 ICP34.5 disables mitochondrial translocation of RIG-I [20].

Moloney leukemia virus 10 protein (MOV10) is a DExD-box RNA helicase consisting of seven highly conserved helicase motifs in its C-terminal region, one of which has a DEAG signature sequence [21]. As a component of cytoplasmic RNA processing centers known as P-bodies [22–24], MOV10 associates with the RNA-induced silencing complex and regulates miRNA-mediated gene repression [25,26]. Additionally MOV10 has been reported to be an ISG [27,28] implying its involvement in antiviral defense. Indeed MOV10 exhibit antiviral activities against a wide range of RNA viruses, including human immunodeficiency virus (HIV), bunyavirus, murine leukemia virus, hepatitis C virus, vesicular stomatitis virus (VSV), influenza A virus, Sendai virus (SeV) and MERS-coronavirus [29–34], except that it appeared to promote hepatitis D virus replication [35]. Current knowledge about the role of MOV10 in DNA virus infection is quite limited. Its restriction of hepatitis B virus infection has been reported [36,37]. However, to our knowledge, the role of MOV10 in infection with herpesviruses, including HSV, has not been investigated.

MOV10’s known role in miRNA-mediated gene silencing implies that it might influence HSV-1 infection because multiple virus and host miRNAs play a role during HSV-1 infection [38,39]. Moreover, one study suggested that the antiviral activity of MOV10 against SeV and VSV was mediated through an RNA sensing pathway [31]. RNA sensing is known to contribute to host defense against HSV-1 invasion too [20,40]. Therefore we hypothesized that MOV10 might regulate HSV-1 infection through miRNA and/or IFN related mechanisms and conducted the following research to test this hypothesis.

## Results

### Induction of MOV10 expression by HSV-1 acute infection in cultured cells and mouse trigeminal ganglia

**Fig 1:**
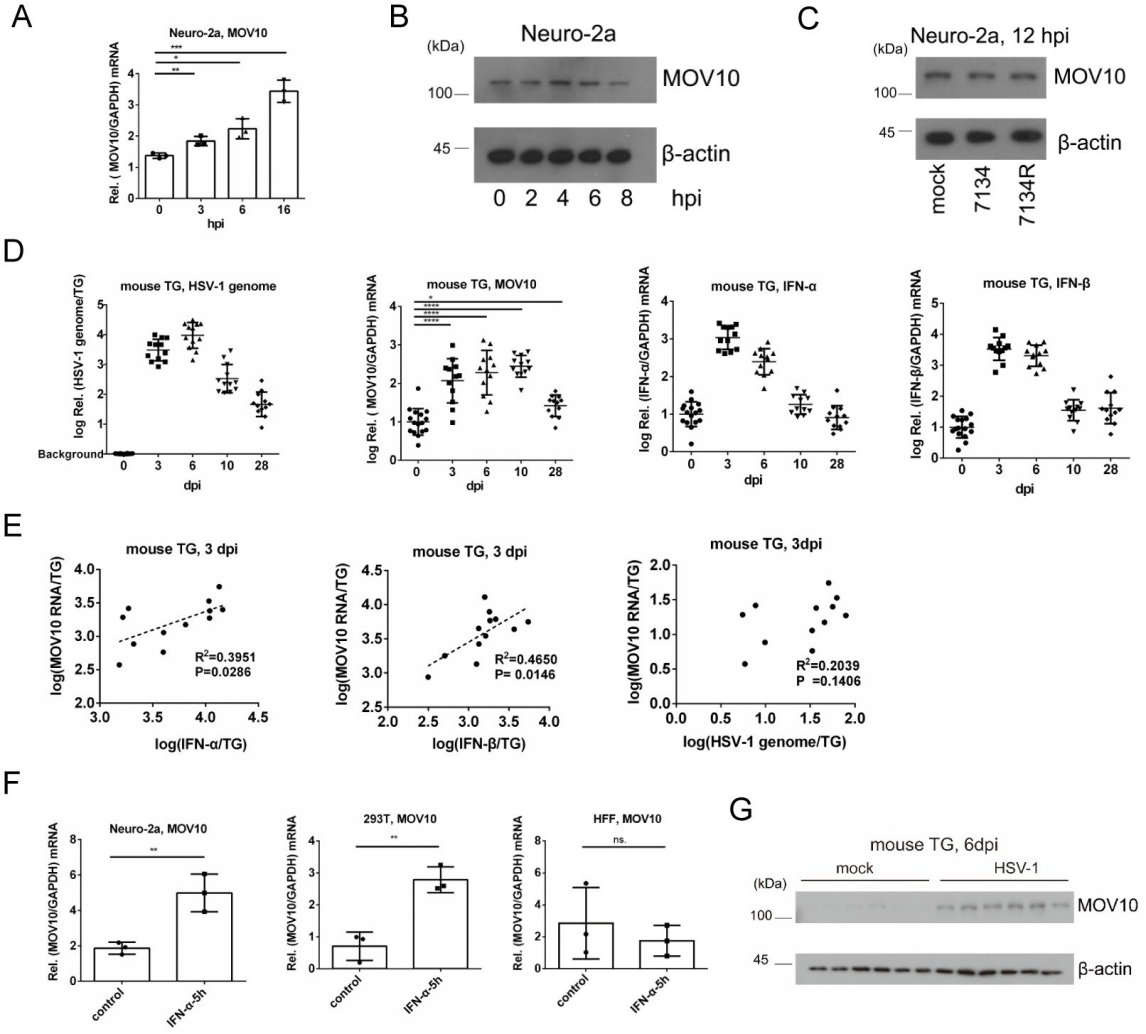
MOV10 expression was induced during HSV-1 acute infection. (A) Neuro-2a cells were infected with HSV-1 (MOI = 1) and then harvested at various times for *Mov10* mRNA quantification by qRT-PCR. (B) Neuro-2a cells were infected with KOS (MOI = 5) and harvested at the indicated times for MOV10 protein analysis by western blots. (C) Neuro-2a cells were mock infected, or infected with 7134 or 7134R (MOI = 5) for 12 h before western blot analysis. (D) Mice were infected on the cornea with 2 x 10^5^ pfu virus per eye. TG were harvested at the indicated days post-infection before qRT-PCR analysis for the DNA or mRNAs indicated at the top of each panel. (E) The levels of *Mov10* mRNA were plotted against the levels of *IFN-α* or *IFN-β* mRNA or viral genome in TG harvested at 3 dpi. R^2^ and p values for each correlation are shown at the right corners of the plots. (F) Neuro-2a, 293T and HFF cells were treated with IFN-α (200 IU/ml) for 5 h before qRT-PCR analysis of *Mov10* mRNA. (G) Mice were infected as in D and TG collected at 6 dpi were analyzed by western blots. Data were analyzed by two-tailed unpaired *t*-tests (F) or one-way ANOVA with Bonferroni’s multiple comparisons (A, D). *, p < 0.05; **, p < 0.01; ***, p < 0.001; ****, p < 0.0001. Data are presented as mean values ± standard deviations (SD).

To explore the anti-HSV-1 potential of MOV10, we first analyzed MOV10 expression during HSV-1 infection. In mouse neuroblastoma Neuro-2a cells, *Mov10* mRNA levels increased gradually over the first few hours after infection with HSV-1 strain KOS at a multiplicity of infection (MOI) of 1 (Fig 1A). HSV-1 strains KOS, F, 17syn+ and Patton all caused comparable increases in *Mov10* mRNA levels at an MOI of 5 at 18 hours post-infection (hpi) (S1A Fig). We used KOS for all of the following experiments. Upregulation of *Mov10* mRNA was also observed in HSV-1 infected human embryonic kidney 293T cells, human foreskin fibroblasts (HFFs) and mouse macrophage Raw 264.7 cells (S1B Fig). However, MOV10 protein levels increased little over the first few hours and seemingly decreased thereafter in Neuro-2a cells (Fig 1B). In Raw264.7 cells, MOV10 protein was upregulated only slightly during infection, and in HFF cells, no upregulation was observed (S1C Fig). These results indicated impairment of MOV10 translation and/or stability during HSV-1 infection. Since the viral E3 ubiquitin ligase ICP0 is known to induce proteosomal degradation of cellular proteins [41], we used an ICP0-null mutant virus 7134, but found that its infection of Neuro-2a cells also caused no upregulation of MOV10 protein (Fig 1C), indicating that MOV10 protein upregulation was blocked by a mechanism independent of ICP0-mediated degradation.

Next we examined MOV10 expression in a mouse model. Following inoculation of KOS onto mouse cornea, we harvested mouse trigeminal ganglia (TG) and quantified both DNA and RNA from the same samples. Viral genomic DNA levels in TG peaked at 3 and 6 days post-infection (dpi) and dropped afterwards until reaching relatively low levels at 28 dpi when latency was fully established (Fig 1D). Meanwhile *Mov10* mRNA was significantly upregulated at 3, 6 and 10 dpi in these TG, with average levels being 1 to 1.5 logs (>10-fold) higher than mock infection (Fig 1D). We note that from 6 to 10 dpi, *Mov10* mRNA levels remained high despite drastic decreases in viral DNA levels. At 28 dpi, *Mov10* mRNA returned to levels that were slightly yet still significantly higher than mock infection, suggesting that while MOV10 upregulation was driven by acute infection, it lasted beyond the acute phase. Considering the known role of MOV10 as an ISG, we also analyzed *IFN-α* and *IFN-β* mRNA levels and observed their increases by 2 and 2.5 logs respectively, on average at 3 dpi and decreases thereafter (Fig 1D). At 3 dpi, *Mov10* mRNA levels correlated significantly with both *IFN-α* and *IFN-β* mRNA levels but not with viral genome levels (Fig 1E), suggesting that MOV10 was more likely induced by the IFN response than by direct viral action. IFN-α treatment stimulated MOV10 expression significantly in Neuro-2a and 293T cells but not in HFF cells indicating that MOV10 might be a conditional ISG (Fig 1F). We then analyzed MOV10 protein in mouse TG by western blots and found that it was barely detected in all the six mock infected TG samples tested but became robustly detectable in all the six HSV-1 infected samples collected at 6 dpi indicating significant upregulation of MOV10 protein (Fig 1G). We note that bulk analyses of the whole tissues probably underestimated the extent to which MOV10 was upregulated in individual infected cells since only a fraction of TG cells are infected. Therefore despite viral antagonism, both MOV10 mRNA and protein were strongly upregulated during HSV-1 acute infection in vivo.

### MOV10 repressed HSV-1 replication and gene expression in multiple cell types

**Fig 2:**
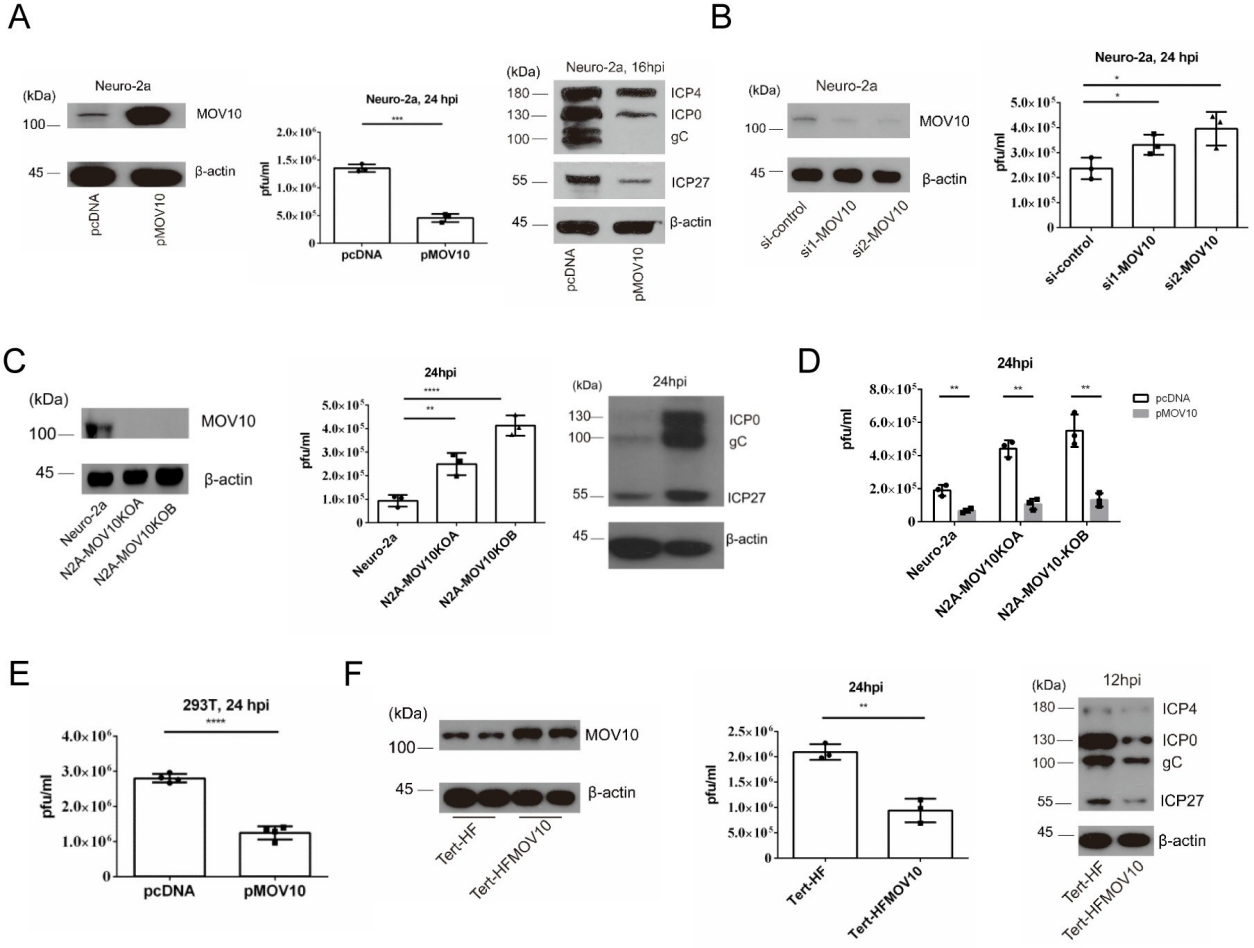
MOV10 repressed HSV-1 replication and gene expression in multiple cell types. (A) Neuro-2a cells were transfected with the indicated plasmid for 24 h at which time the cells were either harvested directly for western blot analysis of MOV10 protein (left) or infected with HSV-1 (MOI = 5) (middle and right). Viral titers were determined by plaque assays at 24 hpi (middle). Viral proteins were analyzed by western blots at 16 hpi (right). (B) Neuro-2a cells were transfected with control siRNA or the indicated siRNA against MOV10 for 36 h, at which time the cells were harvested for western blot analysis of MOV10 protein (left) or infected with HSV-1 (MOI = 1) for 24 h before virus titration. (C) Lack of MOV10 expression in N2A-MOV10KOA and N2A-MOV10KOB cells was verified by western blots (left). After the cells were infected with HSV-1 (MOI = 1) for 24 h, viral titers were determined (middle) and viral proteins were analyzed by western blots (right). (D) The indicated cells were transfected with the indicated plasmid for 24 h before infection with HSV-1 (MOI = 0.2). At 24 hpi, viral titers were measured by plaque assays. (E) 293T cells were transfected with the indicated plasmid for 24 h and then infected with HSV-1 (MOI = 1) for 24 h before viral titer determination. (F) MOV10 expression from MOV10 stably expressing HFF cells and control cells was analyzed by western blots (left). The cells were infected with HSV-1 (MOI = 5) and harvested at 24 hpi for viral titer analysis (middle) or harvested at 12 hpi for western blot analysis (right). Data were analyzed by two-tailed unpaired *t* tests (A, D, E) or one-way ANOVA with Bonferroni’s multiple comparisons (B, C) and are presented as mean values ± SD. *, p < 0.05; **, p < 0.01; ***, p < 0.001; ****, p < 0.0001.

Having observed induction of MOV10 expression during HSV-1 infection, we next investigated the impact of MOV10 on HSV-1 infection. Relative to an empty vector, transfection of a human MOV10 expressing plasmid into Neuro-2a cells significantly decreased HSV-1 yields at 24 hpi and decreased expression of all viral proteins tested (ICP0, ICP4, ICP27 and gC) at 16 hpi (Fig 2A). Accordingly, knockdown of MOV10 by two different small interfering RNAs (siRNAs) both moderately but significantly increased HSV-1 yields relative to a nonspecific control siRNA (Fig 2B). Moreover, two independently generated MOV10 knockout cell lines both showed increased viral yields relative to the parental Neuro-2a cells at 24 hpi, and one of them that we tested showed increased viral protein levels (Fig 2C). Transfection of MOV10 into the knockout cells reduced viral yields to levels lower than those from untransfected Neuro-2a cells indicating that the increases in viral yields were due to MOV10 knockout (Fig 2D). Consistent results were obtained from other cell lines, as transfected MOV10 decreased viral yields in 293T cells (Fig 2E), and Tert-HF fibroblasts stably overexpressing MOV10 showed reduced viral yields and viral protein expression relative to the control cells (Fig 2F). These results demonstrated anti-HSV-1 activity of MOV10 under multiple conditions.

### The N-terminus of MOV10 without the helicase activity was necessary and sufficient for restriction of HSV-1 replication

**Fig 3:**
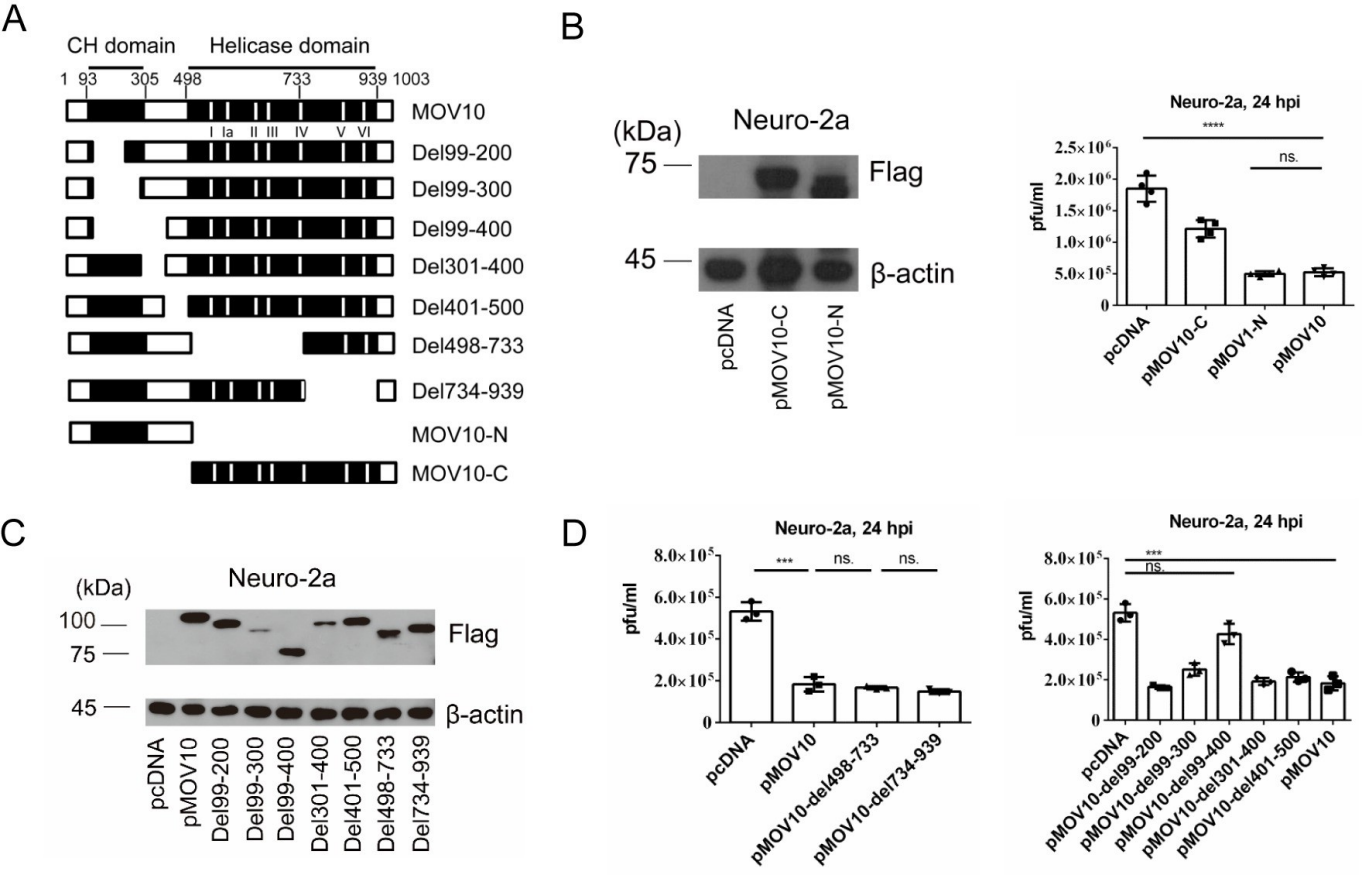
The N-terminus of MOV10 without the helicase activity was necessary and sufficient for MOV10 restriction of HSV-1 replication. (A) Schematic diagram of truncated MOV10 mutants. The numbers indicate amino acid positions. The black boxes to the left and right represent CH and helicase domains, respectively. The positions of the seven helicase motifs are indicated under the first bar. (B) Neuro-2a cells were transfected with the indicated plasmid for 24 h before western blot analysis of Flag-tagged MOV10 mutants (left). At 24 h post-transfection, the cells were infected with HSV-1 (MOI = 5) for 24 before viral titration. (C) Expression of Flag-tagged truncated mutants of MOV10 was verified by western blots. (D) Same as B, except that the indicated plasmids were used. Data were analyzed by one-way ANOVA with Bonferroni’s multiple comparisons and are presented as mean values ± SD. ns, p ≥ 0.05; *, p < 0.05; **, p < 0.01; ***, p < 0.001; ****, p < 0.0001.

The C-terminus of MOV10 contains seven helicase motifs required for its helicase activity and the N-terminus contains a Cys-His-rich (CH) domain (Fig 3A) [29,42]. After transfection of a plasmid expressing only the C-terminus or N-terminus into Neuro-2a cells, the N-terminus retained the ability to suppress HSV-1 replication, whereas the C-terminus only had a moderate effect (Fig 3B) suggesting that the N-terminus was both necessary and sufficient for efficient suppression of HSV-1 replication. Deleting parts of the C-terminus also did not affect the suppression (Fig 3C and 3D), further demonstrating the dispensability of the RNA helicase activity for MOV10 suppression of HSV-1 replication. Using constructs with various sequences deleted at the N-terminus, we found that a region from amino acid residues 99 to 400 was most important for the suppression (Fig 3C and 3D). Thus, MOV10 suppressed HSV-1 replication through a region near the N-terminus without requiring its RNA helicase activity.

### Recombinant viruses expressing MOV10 showed reduced replication in mouse eyes in vivo

**Fig 4:**
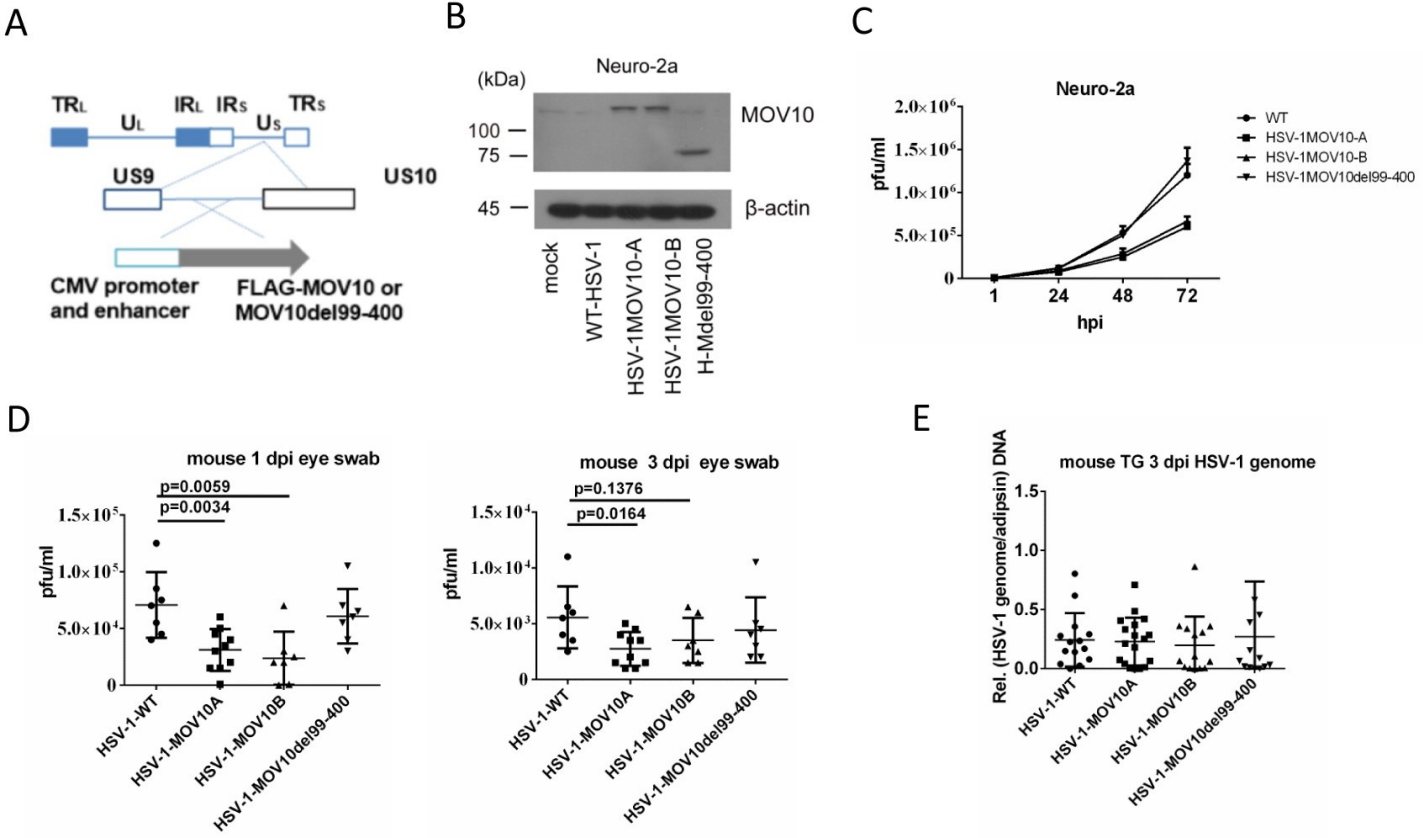
Phenotypes of recombinant viruses expressing MOV10. (A) Schematic diagram of engineered recombinant viruses. Expanded below are the inserted sequences and the location of the insertions. (B) Neuro-2a cells were infected with the indicated viruses at an MOI of 5 for 12 h before western blot analysis of the ectopically expressed proteins by the recombinant viruses using a Flag antibody. (C) Neuro-2a cells were infected with the indicated viruses at an MOI of 0.05 for the indicated times before plaque assay analysis of viral titers. (D) Mice were inoculated on the cornea with 2 × 10^5^ pfu/eye of indicated viruses. Eye swab titers at 1 and 3 dpi were measured by plaque assays. (E) After corneal inoculation as in D, viral genome levels in TG at 5 dpi were measured by qPCR. Data were analyzed by one-way ANOVA with Bonferroni’s multiple comparisons. Data are presented as mean values ± SD.

To examine the effects of MOV10 in vivo, we used recombinant viruses expressing MOV10. We inserted the *Mov10* coding sequence or its del99-400 mutant, with a Flag tag, an upstream CMV promoter and downstream poly(A) signal, into an intergenic region between the *US9* and *US10* genes of the HSV-1 genome using bacterial artificial chromosome (BAC) technology, resulting in two independently constructed MOV10 expressing viruses designated HSV-1MOV10A and HSV-1MOV10B, as well as a control virus designated HSV-1MOV10del99-400 (Fig 4A). Overexpression of MOV10 was confirmed by western blotting after infection of Neuro-2a cells with these recombinant viruses (Fig 4B). Consistent with the transfection-infection results, both HSV-1MOV10A and HSV-1MOV10B displayed reduced replication kinetics relative to the parental wild-type (WT) virus in Neuro-2a cells while HSV1MOV10del99-400 replicated with WT kinetics (Fig 4C). After the same amounts of plaque forming units (pfu) of the recombinant and WT viruses were inoculated into mouse cornea, as confirmed by back titer results, the titers of both HSV-1MOV10A and HSV-1MOV10B, but not HSV-1MOV10del99-400, were significantly lower than WT titers in eye swabs at 1 dpi (Fig 4D). At 3 dpi, the differences in eye swab titers became less pronounced, and unexpectedly, no difference in viral genome levels was observed in TG (Fig 4E). In another experiment, HSV-1MOV10A, but not HSV-1MOV10del99-400 showed lower eye swab titers than WT at 1 and 3 dpi (S2A Fig), but all the viruses showed similar titers in eyes and TG at 5 dpi (S2B Fig). Therefore while the repressive effects of MOV10 were observed at early times in eyes, the effects disappeared later. We speculate that the differential ocular replication at early times might have resulted in different immune responses whose impact might have offset the replication differences. While the effects of MOV10 on HSV-1 replication in TG require further investigation probably using other approaches, these data at least suggested that MOV10 could restrict HSV-1 replication in certain tissues in vivo.

### Specific repression of ICP0 expression in transfected cells and ICP0-independent restriction of HSV-1 replication

Regarding how MOV10 restricted HSV-1 replication, we first considered MOV10’s function in RNA silencing [24,43]. In previous work, we showed that a host neuronal miRNA, miR138, represses viral ICP0 expression by targeting the ICP0 3’ UTR [44]. Therefore we speculated that MOV10 might regulate miR-138 repression of ICP0 expression. We first tested this hypothesis by a luciferase assay. After co-transfection of a MOV10 expressing plasmid and a luciferase construct with ICP0 3’ UTR into Neuro-2a cells where endogenous miR-138 is highly expressed [44], MOV10 had no effect on luciferase expression (S3A Fig) arguing against this hypothesis. Meanwhile, we also performed co-transfection using a plasmid containing the whole ICP0 gene including the 3’ UTR. Surprisingly, MOV10 markedly repressed ICP0 protein expression (S3B Fig). The N-terminus, rather than C-terminus of MOV10, was both necessary and sufficient for this effect (S3C Fig). However, this repression was independent of miR-138 since mutating the miR-138 binding sites or even deleting the whole 3’ UTR did not affect the repression (S3D Fig), in agreement with the above luciferase result. Removing the ICP0 introns also did not affect the repression (S3D Fig). Curiously, the effect appeared to be specific to ICP0 because MOV10 had no effect on the expression of other viral genes tested in such co-transfection assays (S3E Fig). To determine whether this activity accounted for MOV10 suppression of HSV-1 replication, we used an ICP0-null virus, 7134 in comparison with the rescued 7134R virus. Transfected MOV10 reduced yields of both 7134 and 7134R although the effects on 7134 appeared slightly less pronounced (S3F Fig), suggesting that while repressing ICP0 expression might contribute to restriction of HSV-1 replication, it was not required and other mechanisms were important for this restriction.

### MOV10 enhanced type I IFN production during HSV-1 infection and this activity was required for restriction of HSV-1 replication

**Fig 5:**
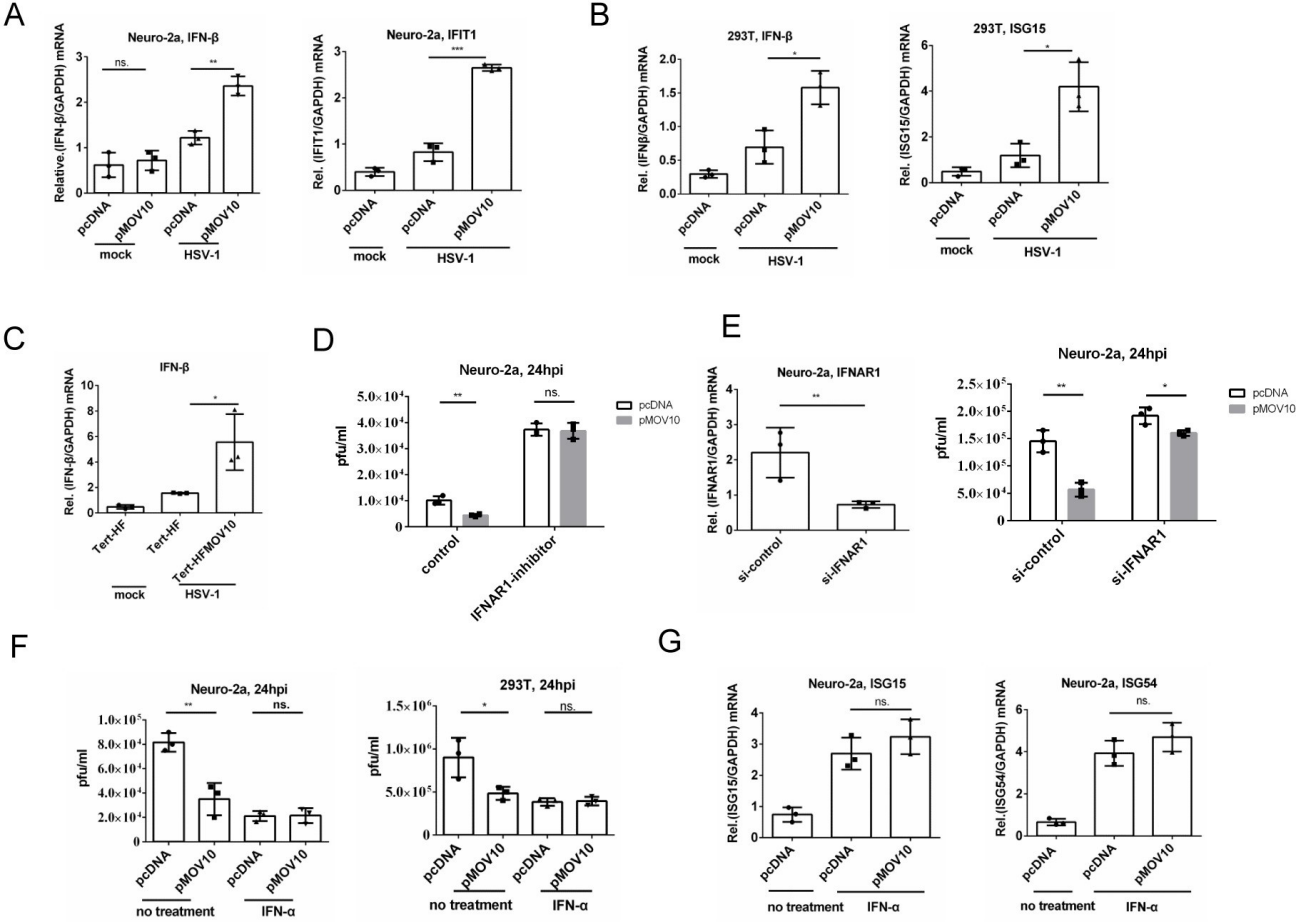
MOV10 restricted HSV-1 replication by enhancing type I IFN expression. (A) Neuro-2a cells were transfected with the indicated plasmid 24 h before mock infection or HSV-1 infection. At 12 hpi, cells were harvested and analyzed by qRT-PCR for the mRNAs indicated at the top. (B) Same as A, but 293T cells were used. (C) Tert-HF and Tert-HFMOV10 cells were mock-infected or infected with HSV-1 (MOI = 0.5). At 12 hpi, cells were harvested for qRT-PCR analysis. (D) Neuro-2a cells were transfected with the indicated plasmid. At 24 h post-transfection, the cells were treated with 2 μM IFNAR1 inhibitor (IFN alpha-IFNAR-IN-1) for 16 h and then infected with HSV-1 (MOI = 1) for 24 h before virus titration. (E) Neuro-2a cells were transfected with control siRNA or siRNA against IFNAR1 for 36 h, at which time the cells were either harvested for qRT-PCR evaluation of knockdown efficiency (left), or infected with HSV-1 (MOI = 1) for another 24 h before virus titration. (F) Neuro-2a (left) or 293T (right) cells were transfected with the indicated plasmid. At 24 h post-transfection, cells were treated with IFN-α (500 IU/ml) for 16 h and then infected with HSV-1 (MOI = 1) for 24 h before virus titration. (G) Neuro-2a cells were transfected with an empty vector or MOV10 expressing plasmid. At 24 h post-transfection, cells were treated with IFN-α (500 IU/ml) for 16 h before quantification of the indicated ISG mRNAs by qRT-PCR. Data were analyzed two-tailed unpaired *t*-tests and are presented as mean values ± SD. ns, p ≥ 0.05; *, p < 0.05; **, p < 0.01; ***, p < 0.001; ****, p < 0.0001.

Given the known role of MOV10 as an ISG [31], we asked whether MOV10 regulated the IFN response to HSV-1 infection. Indeed, despite reduction of viral replication by MOV10 which should usually correlate with reduction of IFN production, overexpressed MOV10 significantly enhanced mRNA levels of *IFN-β* and an ISG, *IFIT1*, in HSV-1 infected Neuro-2a cells while having no effect on their baseline levels in mock infected cells (Fig 5A). Overexpressed MOV10 also upregulated *IFN-β* and *ISG15* mRNAs in HSV-1 infected 293T cells (Fig 5B). Moreover, Tert-HF cells stably overexpressing MOV10 showed higher levels of *IFN-β* mRNA than control cells during HSV-1 infection (Fig 5C). Considering that IFN acts by binding to receptors after being secreted from cells, we took the conditioned media from MOV10-transfected and HSV-1 infected cells and added them to Neuro-2a cells before infection of the Neuro-2a cells. When we compared the medium from MOV10-transfected versus control Neuro-2a cells, the differences were not significant (S4A Fig). However, the medium from Tert-HF-MOV10 cells led to significantly reduced viral yields in Neuro-2a cells relative to the medium from Tert-HF cells (S4B Fig) indicating that secreted protein upregulated by MOV10 could restrict HSV-1 replication under certain conditions. We then assessed the necessity of a type I IFN receptor (IFNAR1) for MOV10 action against HSV-1. Treating Neuro-2a cells with an inhibitor of IFNAR1, IFN alpha-IFNAR-IN-1 [45], ablated the restrictive effect of MOV10 on HSV-1 replication (Fig 5D). Consistently, transfection of an IFNAR1 siRNA greatly attenuated the impact of MOV10 on HSV-1 replication (Fig 5E). These results suggested that either induction of IFN expression or IFN downstream signaling was important for MOV10 restriction of HSV-1 replication. To dissect these two possibilities, we used IFN-α to stimulate IFN downstream signaling. In both Neuro-2a and 293T cells, IFN-α treatment decreased viral replication as expected, but transfected MOV10 could no longer decrease it further relative to the empty vector (Fig 5F). Moreover, while *ISG15* and *ISG54* mRNAs were both upregulated by IFN-α treatment of Neuro-2a cells, MOV10 overexpression had no further effect on these levels in IFN-α treated cells (Fig 5G). These results suggested that MOV10 restricted HSV-1 replication by enhancing the production of type I IFN without influencing its downstream signaling.

### An IKKε-mediated RNA sensing pathway was important for MOV10 restriction of HSV-1 replication

**Fig 6:**
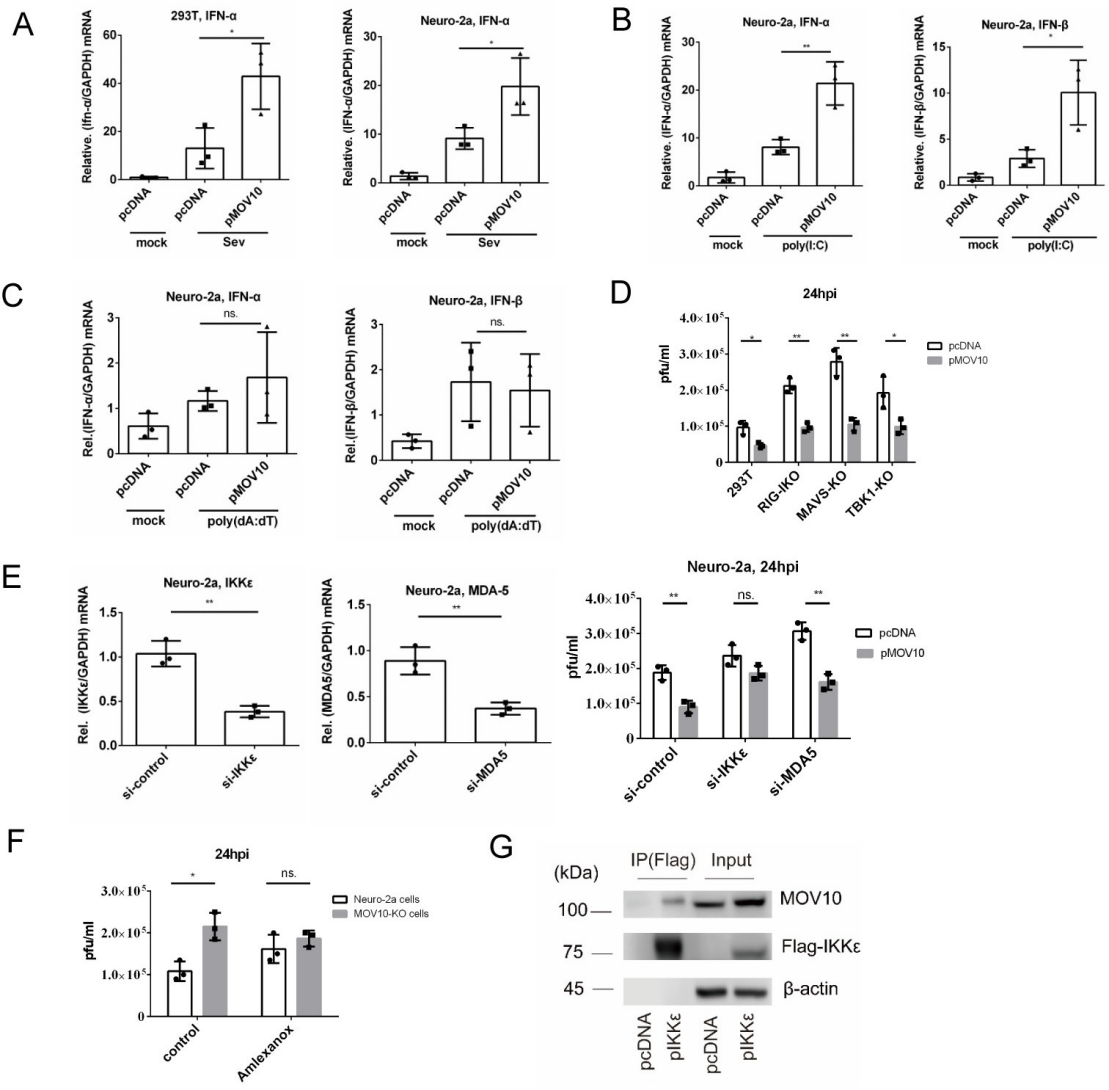
An IKKε-mediated RNA sensing pathway was important for MOV10 restriction of HSV-1 replication. (A-C) 293T or Neuro-2a cells were transfected with the indicated plasmid for 24 h. Then the cells were mock treated, or infected with SeV (MOI = 10) for 12 h (A), or treated with 1 μg/ml poly(I:C) for 16 h (B), or transfected with 1 μg/ml poly(dA:dT) for 6 h (C) before qRT-PCR analysis of the mRNAs indicated at the top. (D) 293T, RIG-IKO, MAVS-KO and TBK1-KO cells were transfected with the indicated plasmid for 24 h and then infected with HSV-1 (MOI = 1) for another 24 h before virus titration. (E) Neuro-2a cells were transfected with control siRNA or siRNA against IKKε or MDA5 for 36 h before qRT-PCR quantification of *IKKε* (left) or *MDA5* (middle) mRNA to evaluate knockdown efficiencies. At 12 h after transfection of these siRNAs, cells were transfected with an empty vector or MOV10 expressing plasmid for 24 h and then infected with HSV-1 (MOI = 1) for another 24 h before virus titration (right). (F) Neuro-2a and N2A-MOV10KO cells were treated with 50 μM Amlexanox for 24 h and then infected with HSV-1 (MOI = 0.5) for another 24 h before virus titration. (G) Neuro-2a cells were co-transfected with a plasmid expressing untagged MOV10 and either an empty vector or a plasmid expressing Flag-tagged IKKε. At 36 h post-transfection, the cells were infected with HSV-1 (MOI = 5). At 12 hpi, the cells were harvested for direct western blot analysis (input) or immunoprecipitated using an anti-Flag antibody before western blot analysis (IP). Data were analyzed two-tailed unpaired *t*-tests and are presented as mean values ± SD. ns, p ≥ 0.05; *, p < 0.05; **, p < 0.01; ***, p < 0.001; ****, p < 0.0001.

To learn about the mechanism by which MOV10 upregulated type I IFN production, we first asked whether MOV10 influenced an RNA or DNA sensing pathway. We recapitulated previous results showing upregulation of IFN-α by transfected MOV10 in SeV infected 293T cells [31], and further showed a similar effect of MOV10 in SeV infected Neuro-2a cells (Fig 6A). Also, MOV10 overexpression in Neuro-2a cells significantly upregulated both *IFN-α* and *IFN-β* mRNAs that had been induced by poly(I:C), a mimic of double-stranded RNA (Fig 6B). However, MOV10 overexpression had no significant effect on *IFN-α* or *IFN-β* mRNA levels in cells transfected with poly(dA:dT), a mimic of double-stranded DNA (Fig 6C), indicating that MOV10 enhanced type I IFN production through an RNA, but not DNA, sensing pathway.

Given these results, we first exploited the available 293T-RIG-IKO, 293T-MAVSKO and 293T-TBK1KO cell lines, which were devoid of RIG-I, MAVS and TBK1, respectively, all of which are important mediators of the RIG-I like receptor RNA sensing pathway. Overexpressed MOV10 suppressed HSV-1 replication in these cells to similar extents to those seen in wild-type 293T cells (Fig 6D) arguing against their importance for this MOV10 function. We then utilized siRNAs to knock down two other major mediators of the pathway, IKKε and MDA5. Knocking down IKKε, but not MDA5, made transfected MOV10 unable to significantly affect HSV-1 replication in Neuro-2a cells (Fig 6E), indicating that IKKε was important for MOV10’s anti-HSV-1 activity. Moreover, addition of Amlexanox, a specific inhibitor of TBK1 and IKKε [46,47], eliminated the difference in HSV-1 replication between N2AMOV10KO and Neuro-2a cells (Fig 6F). Furthermore, MOV10 co-immunoprecipitated with ectopically expressed IKKε in HSV-1 infected Neuro-2a cells (Fig 6G). These results suggested that MOV10 restricted HSV-1 replication through an IKKε-mediated RNA sensing pathway of IFN induction independently of RIG-I, MDA-5 and MAVS.

**Fig 7:**
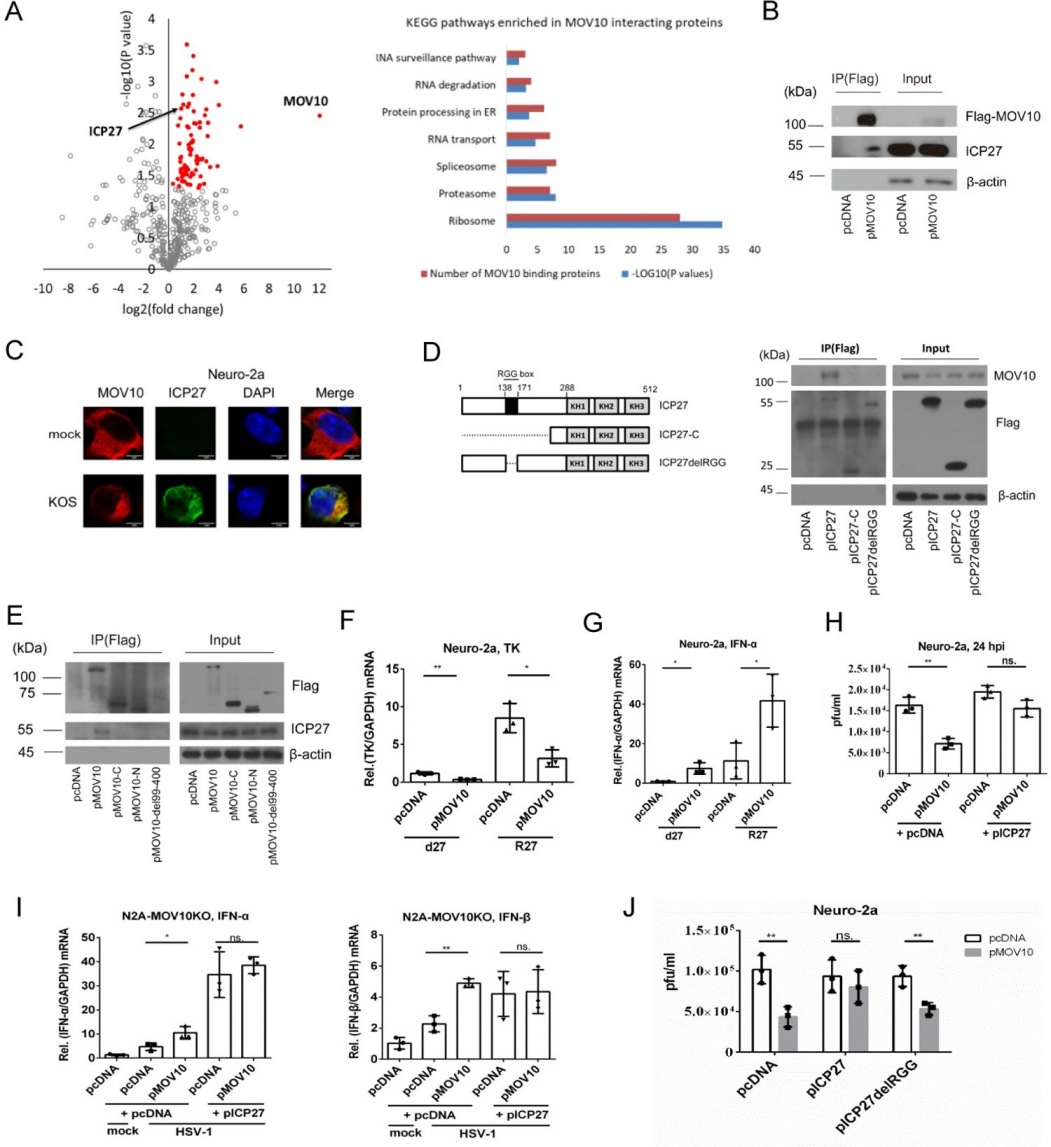
HSV-1 protein ICP27 interacted with MOV10 to antagonize MOV10 restriction of HSV-1 replication. (A) Left, volcano plot for the differences between protein levels in the MOV10 specific pulldown group and control pulldown group as analyzed by LC-MS/MS. Red dots represent candidate interacting proteins according to the selection criteria of *p* < 0.05 (*t*-test). Right, enriched pathways for the interacting proteins as determined by KEGG analysis with the P values and number of proteins displayed. (B) Neuro-2a cells were transfected with an empty vector or a Flag-tagged MOV10 expressing plasmid 24 h before infection with HSV-1 (MOI = 5). The cells were harvested at 12 hpi, at which time they were either directly analyzed by western blots (input) or immunoprecipitated using a Flag tag antibody before western blotting (IP) for the proteins indicated to the right. (C) Neuro-2a cells were transfected with the MOV10 expressing plasmid. At 24 h post-transfection, the cells were mock infected or infected with HSV-1 (MOI = 5) for 12 h and then analyzed using confocal microscopy after staining with antibodies against the MOV10 and ICP27. Scale bar, 5 μm. (D) Left, schematic diagram of truncated ICP27 mutants. The numbers indicate amino acid positions. The black box represents the RGG box. Right, Neuro-2a cells were transfected with the indicated plasmid expressing ICP27 or its truncated mutants. Cells were harvested at 24 h post-transfection for direct western blot analysis (input) or immunoprecipitated using an anti-Flag antibody before western blot analysis (IP). (E) Neuro-2a cells were transfected with the indicated plasmid expressing MOV10 or its truncated mutants. At 24 h post-transfection, cells were infected with HSV-1 (MOI = 5). At 12 hpi, cells were harvested for direct western blot analysis (input) or immunoprecipitated using an anti-Flag antibody before western blot analysis (IP). (F) Neuro-2a cells were transfected with the MOV10 expressing or control plasmid 24 h before infection with d27 or R27 virus (MOI = 1). Cells were harvested at 12 hpi and analyzed by qRT-PCR for *TK* mRNA. (G) Neuro-2a cells were transfected with the indicated plasmid 24 h before infection with d27 or R27 virus (MOI = 2). Cells were harvested at 24 hpi and analyzed by qRT-PCR for *IFN-α* mRNA. (H) Neuro-2a cells were co-transfected with an empty vector or MOV10 expressing plasmid, together with an empty vector or ICP27 expressing plasmid 24 h before infection with HSV-1 (MOI = 0.5). The cells were harvested at 24 hpi for virus titration. (I) Same as H, except that N2A-MOV10KO cells were used and harvested at 12 hpi for qRT-PCR quantification of the indicated IFN mRNAs. (J) Same as H except that the indicated plasmids were used for transfection. Data were analyzed two-tailed unpaired *t*-tests and are presented as mean values ± SD. ns, p ≥ 0.05; *, p < 0.05; **, p < 0.01.

### HSV-1 protein ICP27 interacted with MOV10 and its overexpression antagonized MOV10’s anti-HSV-1 activity in an RGG box dependent manner

The suppressive role of MOV10 led to the question of whether HSV-1 had counter measures. To address this, we first searched for proteins that interacted with MOV10. Following transfection of Neuro-2a cells with MOV10 and infection with KOS, we immunoprecipitated MOV10 and then analyzed the interacting proteome by liquid chromatography (LC)-mass spectrometry (MS)/MS. No peptide from IKKε was detected which might be due to limited sensitivity and low IKKε expression. Nevertheless, peptides from numerous viral and host proteins were detected. We combined proteins exclusively identified in the MOV10 pulldown group as well as those showing significantly different peptide counts between the specific pulldown and control groups (P < 0.05) (Fig 7A), resulting in 101 candidate interacting proteins (S1 Table). KEGG enrichment analysis showed that these proteins were functionally enriched in RNA related pathways with the ribosome pathway showing the highest enrichment (Fig 7A). Interestingly, ICP27 was the only viral protein among the candidates. We validated the interaction between MOV10 and ICP27 by western blotting after pulling down transfected MOV10 in HSV-1 infected Neuro-2a cells (Fig 7B). ICP27 expressed during HSV-1 infection also co-localized with transfected MOV10 in Neuro-2a cells in an immunofluorescence assay (Fig 7C). Moreover, we confirmed that endogenous MOV10 could co-immunoprecipitate with transfected ICP27 (Fig 7D). Using plasmids expressing truncated MOV10 we found that the N-terminus, C-terminus and the aa99-400 region were all required for interaction with ICP27 (Fig 7E) indicating that this interaction might involve multiple domains of MOV10. Using plasmids expressing truncated ICP27 mutants we found that the N terminus, especially the RGG box motif of ICP27, was required for interaction with MOV10 (Fig 7D). To test whether ICP27 could regulate MOV10’s antiviral activity, we first utilized an ICP27 deletion mutant virus, d27 in comparison with its rescued virus R27. The d27 mutant was unable to produce infectious virus, but analysis of the viral thymidine kinase (TK) mRNA showed similar repressive effects of transfected MOV10 in d27 and R27 infected Neuro-2a cells (Fig 7F). Transfected MOV10 also increased *IFN-α* mRNA levels in both d27 and R27 infected cells to comparable extents (Fig 7G). However, after co-transfection with ICP27 in Neuro-2a cells, MOV10 could no longer significantly affect HSV-1 replication (Fig 7H) or type I IFN expression during HSV-1 infection (Fig 7I) although the effects remained when MOV10 was co-transfected with an empty vector or a plasmid expressing the RGG-box deletion mutant of ICP27 (Fig 7J) suggesting that ICP27 could potentially antagonize MOV10’s anti-HSV-1 activity in an RGG-box dependent manner.

## Discussion

In this study, we established MOV10 as a novel restriction factor for HSV-1 lytic infection. Following its induction by HSV-1 acute infection, MOV10 induced type I IFN production through an IKKε-mediated RNA sensing pathway. Meanwhile viral ICP27 bound to MOV10 and potentially antagonized its anti-HSV-1 activity.

Like many antiviral proteins, MOV10 expression was induced during HSV-1 infection. Increases in *Mov10* mRNA levels of >10 -fold in whole TG were remarkable considering that only limited numbers of cells were infected in such tissues. We also note that robust MOV10 upregulation in mouse TG lasted from day 3 to at least day 10, which should be a period long enough for MOV10 to have an impact. The role of MOV10 as an ISG has been suggested by several studies. A previous analysis of more than ten published data sets identified MOV10 as one of 389 ISGs upregulated by type I IFN [27], and transcriptome analyses identified MOV10 as one of 62 ISGs conserved in ten vertebrate species [28]. We showed that MOV10 expression was induced by IFN-α treatment in Neuro-2a and 293T cells but not in HFF cells indicating that the role of MOV10 as an ISG might be cell-type dependent, which was consistent with another study showing that MOV10 was only weakly induced by type I IFN in certain cell types [30]. Nevertheless we argue that MOV10 upregulation in vivo likely resulted from the immune response instead of direct viral action given the correlation of levels of *Mov10* mRNA with *IFN-α* and *IFN-β* mRNAs but not with viral DNA during acute infection.

This study adds HSV-1 to a list of viruses reportedly targeted by MOV10 for restriction. Diverse mechanisms are used by MOV10 against different viruses. In many cases, the inhibitory effects of MOV10 require direct interactions with viral proteins, such as the interactions of MOV10 with HIV Gag protein [29], influenza virus nucleoprotein [33,48], MERS-CoV nucleocapsid protein [34], and bunyavirus N protein [30]. In contrast to these mechanisms, repression of HSV-1 replication by MOV10 does not appear to rely on a direct interaction with a viral protein. Instead, the only interaction of MOV10 with an HSV-1 protein that we could identify, i.e., with ICP27, appears to counteract MOV10’s activity. There are also similarities between HSV-1 and other viruses in how they are regulated by MOV10. MOV10’s antiviral activities against most viruses, including HSV-1 shown here, are independent of its helicase activity [30,32,33,49], with only its activity against MERS-coronavirus being an exception [34]. Importantly, our results suggesting that MOV10 restricts HSV-1 replication through an IKKε-mediated RNA sensing pathway agree well with a previous report that MOV10 restricted replication of some RNA viruses via this pathway [31] indicating that MOV10 might use common mechanisms to regulate infection with different viruses on top of the unique mechanisms for individual viruses. Interestingly, given the likely role of MOV10 as an ISG itself, its promotion of the IFN response should provide a positive feedback loop for innate immunity.

MOV10 was initially identified as an Argonaut binding protein required for miRNA-mediated mRNA cleavage [22]. Its role in miRNA mediated gene regulation was corroborated by multiple studies although its effects on individual RNAs are context dependent [24,50]. However, we find no evidence that MOV10 regulates miR-138 repression of ICP0 expression. Instead, MOV10 could repress ICP0 expression independently of miR-138 by unknown mechanisms in transfected cells and this repression was not required for MOV10 restriction of HSV-1 replication in Neuro-2a cells. Nevertheless, it remains possible that MOV10 repression of ICP0 expression has greater impact in other situations such as establishment of latency where ICP0 is more critical [51]. Also, our results do not exclude the possibility that MOV10 regulates functions of other viral or host miRNAs that may influence HSV-1 infection.

In the face of host restriction, viruses often evolve evasive strategies. The lack of strong MOV10 protein upregulation despite significant mRNA upregulation in HSV-1 infected cells indicates viral interference with MOV10 protein synthesis or stability. While this mechanism might be nonspecific, the interaction between MOV10 and ICP27 was specific. Overexpressed ICP27 eliminated the anti-HSV-1 effects of MOV10 indicating that ICP27 might bind to MOV10 to interfere with its activity. Moreover, the inability of the ICP27 RGG-box deletion mutant to interact with MOV10 coincided with its inability to antagonize the anti-HSV-1 activity of MOV10. Although we saw no meaningful difference between d27 and R27 in MOV10’s effects on viral gene expression and IFN-α production, these experiments were complicated by the fact that efficient viral gene expression and type I IFN production also depended on ICP27 (Fig 7D and 7E). Therefore we argue that ICP27 expressed by HSV-1 should at least partially confer resistance to MOV10’s anti-HSV-1 activity especially under circumstances where ICP27 expression is high. ICP27 is a multifunctional protein mainly known for its roles in regulating RNA splicing and nuclear export [52–54]. However, it has also been reported to counteract induction of cytokine expression by HSV-1 [55], to inhibit type I IFN production through the cGAS-STING-TBK1 pathway [56], and to inhibit IFN downstream signaling though the STAT/Jak pathway [57]. Therefore ICP27 appears to be a multifaceted regulator of innate immunity too.

The establishment of MOV10 as a host restriction factor against HSV-1 raises the interesting question of whether other herpesviruses are regulated by MOV10. Given its regulation of an RNA sensing pathway that may influence both RNA and DNA viruses, MOV10’s potential role as a broad antiviral factor deserves consideration. More work is needed to further understand its role in viral infection.

## Materials and methods

### Ethics statement

Animal experiments were performed in accordance to the guidelines for the Care and Use of Medical Laboratory Animals (Ministry of Health, China) and approved by the Animal Research Committee of Zhejiang University.

### Cells

Neuro-2a, 293T, Vero and U2OS cells were obtained from American Type Culture Collection and maintained as previously described [58,59]. V27 cells (Vero cells stably expressing ICP27) [60] and Tert-HF cells [60] were generous gifts from David M. Knipe at Harvard Medical School and maintained as described. 293T-RIGIKO, 293T-MAVSKO and 293T-TBK1KO cells were generous gifts from Nan Qi at Zhejiang University of Technology [61] and maintained like 293T cells. Raw264.7 cells were a generous gift from Xiaojian Wang at Zhejiang University and maintained in Dulbecco’s Modified Eagle Medium supplemented with 10% fetal bovine serum at 37°C in a humidified chamber containing 5% CO_2_.

### Viruses

HSV-1 strains KOS, 17syn+, F and Patton, and KOS derivatives 7134 (ICP0 deletion virus) and 7134R (ICP0 rescued virus) [62] were generous gifts from Donald M. Coen at Harvard Medical School. d27 and R27 [63] viruses were generous gifts from David M. Knipe at Harvard Medical School. SeV was a generous gift from Xiaojian Wang at Zhejiang University. Virus propagation, infection and titration by plaque assays were performed according to previous protocols [58,64].

### Plasmids

FLAG-HA-pcDNA3.1 (#52535) was purchased from Addgene and renamed pcDNA here. Based on this, we constructed plasmids expressing MOV10 and IKKε with Flag-HA tags. The ICP0-WT plasmid (pRS-1) and ICP0-M138 plasmids were previously described [44,65]. To construct ICP0no3’UTR plasmid, the sequence between MluI and stop codon of pRS-1 was amplified and inserted between MluI and HindIII sites of pRS-1. As for ICP0nointron plasmid, ICP0 CDS fragment was amplified and then inserted between NcoI and MluI sites of pRS-1. To construct plasmids expressing truncated MOV10 mutants, we amplified the truncated Mov10 gene sequences by PCR and cloned them into the FLAG-HA-pcDNA3.1 vector using the Site-Directed Mutagenesis kit (#E0552S) purchased from New England Biolabs. Human MOV10 expressing sequences were amplified and inserted between the BamHI and XbaI restriction sites of pLVX-puro (Addgene) plasmid to construct pLVX-puroMOV10 plasmid for lentivirus production. Plasmids expressing MOV10 N- terminus (amino acids 1–495) and MOV10 C-terminus (amino acids 496–1003) were generously provided by Professor Zhiwei Wu (Nanjing University) [49]. Renilla luciferase plasmid and IFN-β promoter reporter plasmid were kindly provided by Chunfu Zheng at Fujian Medical University. The sequences of all primers used are listed in S2 Table.

### qPCR and qRT-PCR

DNA and RNA were isolated using a DNA/RNA Isolation Kit (TIANGEN) and reverse transcribed using a miRNA 1st Strand cDNA Synthesis kit (Vazyme). PCR was performed in ChamQ Universal SYBR qPCR Master mix (Vazyme) according to the manufacturer’s instructions in a QuantStudio3 realtime PCR machine (Applied Biosystems). GAPDH mRNA was used to normalize gene transcript levels. The primers used for qPCR and qRT-PCR are listed in S3 Table.

### Mouse procedures

Six-week male CD-1 (ICR) mice were purchased from Shanghai Laboratory Animals Center. For HSV-1 infection, mice were anesthetized by intraperitoneal injection of 0.4 ml of a mixture consist of 500 ug/ml xylazine hydrochloride (Sigma, X1251) plus 4 mg/ml pentobarbital sodium (Solarbio) in sterile saline. Then 2 x 10^5^ pfu of virus was added onto each scarified cornea in 3 μl of PBS. For TG acquisition, mice were euthanized by cervical dislocation while under anesthesia with isoflurane and TG were collected into the lysis buffer and placed on dry ice before RNA extraction and placed into -80°C as described previously [59]. For western blot analysis, we homogenized TG in lysis buffer (Solarbio, R0020) and extracted protein according to the manufacturer’s protocol. For determination of viral titers in TG, TG were homogenized in cell culture media for titer determination. For eye swab collection, mice were anesthetized in an induction chamber with isoflurane (3% in oxygen 0.5 ml/min) using a V1 Table Top anesthesia machine (Colonial Medical Supply). Both eyes of each mouse were swabbed with cotton-tipped applicators, suspended in 1 ml of cell culture medium.

### Transfection and cell treatment

Cells were transfected with plasmids or siRNAs using Lipofectamine 3000 (Invitrogen) following the manufacturer’s protocol. Cells were plated in 24-well plates at a density of 1.5 x 10^5^ cells/well and transfected with 200 ng plasmids per well for 24 h or 20 nM siRNA per well for 36 h. For co-transfection, cells were transfected with the indicated amounts of two plasmids for 24 h. The target sequences in mouse MOV10 for siRNAs were as follows: si1-MOV10: 5’-GGTACTAACCCTACGGCTT-3’; si2-MOV10: 5’-GCTATGAACTCCACATCTA-3’; si-IKKε: 5’-GGAGGCTGAATCACCAGAA-3’; si-MDA5: 5’- GCACUAUUCCAAGAACUAATT-3’. Amlexanox was purchased from Selleck (S3648). IFN alpha-IFNAR-IN-1 (HY-12836A), was purchased from MedChemExpress. Poly(I:C) and poly(dA:dT) were kind gifts from Chunfu Zheng at Fujian Medical University. Neuro-2a cells were treated with 1 μg/ml poly(I:C) for 16 h before analyzed by qRT-PCR. Poly(dA:dT) were transfected using Lipofectamine 3000 at a concentration of 1 μg/ml. For inhibition of transcription, actinomycin D (MedChemExpress, HY-17559) was used at a final concentration of 10 μg/ml.

### Western blot analysis

Western blotting was performed as previously described [66]. The following primary antibodies and dilutions were used: MOV10 (ab189919) and ICP4 (ab6514) antibody were purchased from Abcam. ICP27 antibody (1113) was purchased from Virusys. Actin antibody (Ac026) and IKKi (A0244) antibody were made by Abclonal. gC antibody (10-H25A) was purchased from Fitzgerald. Flag antibody (M185-3L) was purchased from MBL. Goat anti-mouse and goat anti-rabbit antibodies (SouthernBiotech, 1030–05, 4030–05) were used as secondary antibodies with a dilution of 1:2000.

### Immunofluorescence assay

Neuro-2a cells plated on coverslips were mock-transfected or transfected with 400 ng/ml MOV10 plasmid or control plasmid for 24 h. Then cells were infected with HSV-1 (MOI = 5) for 12 h before being fixed in 4% paraformaldehyde (PFA) for 10 min. After being washed with PBS for 5 min, the cells were treated with 0.1% Triton X-100 in phosphate-buffered saline (PBS) for 10 min and washed with PBS 3 times for 5 min each. Then the cells were blocked with 1% bovine serum albumin in PBS for 1 h at room temperature, before incubation with a primary antibody at 4°C overnight. The cells were then washed 3 times for 10 min each and incubated with a secondary antibody for 1 h. Goat anti-mouse Alexa-488 (Abcam, ab150117) and Alexa555-anti-rabbit IgG (Cell Signaling, 4413S) antibodies were used. Next, 2 ug/ml DAPI was added 10 min before four washes for 10 min each. The coverslips were mounted onto microscope slides with anti-fading reagent, Fluoromount-G (0100–01), purchased from SouthernBiotech. Images were acquired on an OLYMPUS IX83-FV3000 microscope with a 100x magnification.

### Immunoprecipitation

Neuro-2a cells were transfected with the indicated plasmid for 24 h before infection with HSV-1 at an MOI of 10. At 6 hpi, cells were lysed in lysis buffer (50 mM HEPES-KOH [pH 7.4], 1% Triton X-100, 150 mM NaCl, 10% glycerol, and 2 mM EDTA plus one Complete EDTA-free protease inhibitor tablet [Roche] per 50 ml). IP was performed according to a previous protocol [67]. Anti-FLAG beads (Sigma-Aldrich) were used to specifically pull down the targeted protein and mouse IgG-agarose (Sigma-Aldrich) was used as a control to pull down nonspecific protein. 50 μL 1 x SDS loading buffer was added to elute proteins before Western-blot analysis.

### LC-MS/MS

Immunoprecipitated proteins were eluted in 200 μL of elution buffer (0.1 M Glycine, pH 2.8) and neutralized with 25 μL of neutralization buffer (1 M Tris, pH 8.5). Proteins were then reduced with 10 mM dithiothreitol (DTT) for 45 min at 30°C, alkylated with 30 mM iodoacetamide (IAA) for 30 min at room temperature in the dark, and then precipitated by acetone. Proteins were digested by trypsin (Promega) at a ratio of 1:50 (trypsin:protein) in 50 mM ammonium bicarbonate (ABC) at 37°C overnight. Tryptic peptides were acidified by formic acid (FA) and was desalted by 50mg C18 Sep-Pak SPE (Waters) and dried by vacuum lyophilizer (LABCONCO) before LC-MS/MS analysis. Peptides from each sample were subject to nanoLC-MS/MS analysis on an UltiMate 3000 RSLCnano system (Thermo Scientific) coupled to a Q Exactive HFX (Thermo Scientific) mass spectrometry. Peptide were trapped by an Acclaim PepMap 100 reversed-phase pre-column (20 mm X 75 μm, 5 μm, Thermo Scientific) and then separated by an Acclaim PepMap 100 reversed-phase column (250 mm X 75 μm, 2 μm, Thermo Scientific#164941) at the flow rate of 400 nL/min. Solvent A is 2% ACN, 0.1% FA and solvent B is 98% ACN, 0.1% FA. Gradient elution was performed at 50°C using linear gradients of 120 min: 1–4 min with 3% (v/v) of B, 4–6 min from 3% to 5% (v/v) B, 6–70 min from 5% to 15% (v/v) B, 70–90 min from 15% to 30% (v/v) B, 90–100 min from 30% to 80% (v/v) B, 100–110 min with 80% (v/v) B, 110–120 min with 3% (v/v) B. The eluted peptides were analyzed by acquiring MS spectra at the resolution of 120,000 full width at half maximum (FWHM) with a mass range of 300–1800 m/z and an automatic gain control target of 1E6. The top 20 precursors were then fragmented by higher-energy collisional dissociation with 30% normalized collisional energy and MS2 spectra were acquired at the resolution of 12,000 FWHM.

Raw MS data were loaded into MaxQuant software and searched against mouse and HSV-1 UniProtKB protein sequences supplemented with reversed sequences for target-decoy search. Label-free iBAQ algorithm was configured for quantitation analysis. Trypsin was set as digestion mode with 2 maximum missed cleavage sites. Variable modifications included oxidation (M) (+15.99491 Da) and acetyl (protein N-term) (+42.01056 Da), and carbamidomethyl (C) (+57.02146 Da) was set as the fixed modification. MaxQuant default setting was used for all other parameters. Peptide and protein identification were both filtered by false discovery rate (FDR) < 1%.

Potential MOV10 interacting proteins were selected by enriched proteins in the MOV10 IP samples with p < 0.05 (*t-*test) as compared to controls samples, or proteins exclusively identified in all 3 MOV10 samples. Proteins with fewer than 2 unique peptides were discarded. Biological functions or pathways associated with MOV10 interactors were revealed by functional annotation enrichment analysis in KOBAS 3 (http://kobas.cbi.pku.edu.cn) against the GO or KEGG database. Fisher’s exact tests and the Benjamini and Hochberg’s FDR < 0.01 method were used to identify significantly enriched terms.

### Lentivirus production

Lentivirus production and transduction were performed as previously described [59]. Briefly, 293T cells were plated at a density of 8 x 10^6^ cells/plate in 100 mm plates 24 h before co-transfection with 4.3 ug pLVX-puroMOV10 plasmid, 3.8 ug psPAX2 and 1.9 ug pMD2G plasmids using Lipofectamine 3000 (Invitrogen). The medium was replaced with complete growth medium at 24 h post-transfection. The supernatant containing lentivirus was collected at 48 h post-transfection, at which time fresh medium was supplied. At 72 h post-transfection, the supernatant was collected again and combined with that collected on the previous day. The combined supernatant was spun at 2000 rpm for 10 min to remove cells in the pellet and filtered with 0.45 μm syringe filter (Pall). Lentivirus was concentrated by centrifugation at 35,000 rpm for 90 min and the pellet was resuspended with 0.5 ml of growth medium.

### Construction of Tert-HF-MOV10 cells and N2A-MOV10KO cells

Tert-HF-MOV10 cells were constructed by lentivirus transduction as previously described [44]. Briefly, lentivirus expressing MOV10 was added to Tert-HF cells in the presence of 8 μg/ml hexadimethrine bromide (Sigma). On the next day, the medium was replaced with fresh medium containing 2 μg/ml puromycin. Puromycin-resistant cells were selected for 10–14 days during which the medium was changed every 2 days. MOV10 stable expression was confirmed by Western blot analysis.

The N2AMOV10KO cell line was generated according to a method [68] described previously and the target sequence we used was 5’-TTTTCTGGCCGAACGTGGACTGG-3’. Synthetic oligo nucleotides for cloning small guide RNAs (sgRNAs) were designed as described and cloned into the PX459 vector (Addgene) that expresses Cas9. Neuro-2a cells were plated at a density of 1.5 X 10^5^ cells per well in a 24-well plate and transfected with 200 ng of the sgRNA expressing plasmid. The next day, the supernatant was replaced with fresh media and the cells were transfected with the same plasmid once more. After selection with 2 μg/ml puromycin for 2 days, cells were analyzed by a T7E1 assay for verification of genetic editing. Correctly edited cells were limitedly diluted to obtain signal colonies in 96-well plates. When the single colonies were grown, they were trypsinized and transferred to a 12-well plate for expansion. When cells became confluent, some cells were used for DNA extraction using the Tissue & Cell Genomic DNA Purification kit (GeneMark), PCR amplification and sequencing. Other cells were further expanded until they were stably maintained.

### Statistical analysis

Statistical analysis was performed using GraphPad Prism version 7.00 for Windows, GraphPad Software, La Jolla California USA. Unpaired two-tailed *t* -tests were used for comparing two groups. One-way ANOVA with Bonferroni’s multiple comparisons tests were used for comparing multiple groups. Data were presented as means ± standard deviations (SDs). A difference was considered significant when the *P*-value was *<* 0.05.

## Supporting information

S1 Fig MOV10 expression during HSV-1 infection. (A) Neuro-2a cells were infected with the HSV-1 strains as indicated (MOI = 5) and harvested at 18 hpi for *Mov10* mRNA quantification by qRT-PCR. (B) 293T (left), HFF (middle) and Raw264.7 (right) cells were infected with HSV-1 strain KOS (MOI = 5) and harvested at 12 hpi for *Mov10* mRNA quantification by qRT-PCR. (C) Raw264.7 (left) and HFF (right) cells were infected with KOS (MOI = 5) and harvested at the indicated times for MOV10 protein analysis by western blots. Data were analyzed by one-way ANOVA with Bonferroni’s multiple comparisons (A) or two-tailed unpaired *t* tests (B) and are presented as mean values ± SD. *, p < 0.05; **, p < 0.01; ***, p < 0.001; ****, p < 0.0001.
(TIF)

S2 Fig Recombinant viruses expressing MOV10 showed reduced HSV-1 replication in mouse eyes in vivo. (A) Mice were inoculated on the cornea with 2 × 10^5^ pfu/eye of indicated viruses. Eye swab viral titers of WT, HSV-1-MOV10A and HSV-1-MOV10del99-400 were measured at 1 (left), 3 (middle) and 5 (right) dpi. (B) At 5 dpi, mouse TG were collected to determine titers of the indicated viruses. Data were analyzed by one-way ANOVA with Bonferroni’s multiple comparisons. Data are presented as mean values ± standard deviations (SD).
(TIF)

S3 Fig Repression of ICP0 expression and ICP0-independent suppression of HSV-1 replication by MOV10. (A) Neuro-2a cells in a 24-well plate were transfected with 200 ng of pcDNA or pMOV10 and 40 ng of the empty luciferase contruct psiCheck-2 or a construct with the ICP0 3’ UTR. Luciferase was measure at 48 h post-transfection. (B) Neuro-2a cells were transfected with 150 ng of an empty vector or MOV10 expressing plasmid, together with 50 ng of an ICP0 expressing plasmid. The cells were harvested at indicated times for western blot analysis of MOV10 and ICP0. (C) Same as B except that different plasmids were used and the cells were harvested at 48 h post-transfection. (D) Left, diagram of different ICP0 expressing constructs. Blue boxes represent exons. Small red boxes represent miR-138 binding sites in the ICP0 3’ UTR. Middle and right, co-transfection was performed as in B except that different ICP0-expressing constructs as indicated at the top were used and the cells were harvested at 48 h post-transfection. (E) Same as B, but plasmids expressing different viral genes were used for co-transfection with the MOV10 expressing plasmid and the corresponding viral proteins were analyzed. (F) Neuro-2a cells were transfected with an empty vector or a MOV10 expressing plasmid for 24 h before infection with 7134 or 7134R virus (MOI = 0.1). Cells were harvested at 48 hpi for virus titration. Data were analyzed by two-way ANOVA with Bonferroni’s multiple comparisons and are presented as mean values ± SD. *, p < 0.05; **, p < 0.01; ***, p < 0.001; ****, p < 0.0001.
(TIF)

S4 Fig Effects of pretreatment of Neuro-2a cells with conditioned media on HSV-1 replication. (A) Neuro-2a cells were transfected with indicated plasmid for 24 h and infected with HSV-1 (MOI = 0.5) for 12 h before collection of the supernatants. Neuro-2a cells were pretreated with these supernatants for 12 h and then infected with HSV-1 (MOI = 0.2). Viral titers were determined by plaque assays at 42 hpi. (B) Tert-HF and Tert-HFMOV10 cells were infected with HSV-1 (MOI = 0.5) for 12 h before collection of the supernatants. Neuro-2a cells were pretreated with these supernatants for 12 h and then infected with HSV-1 (MOI = 0.5). Titers were measured at 24 hpi by plaque assays.
(TIF)

S1 Table List of proteins identified by the MOV10 interactome analysis. Original peptide counts, fold changes and P values for comparisons between the MOV10 pulldown group and control group are shown.
(XLSX)

S2 Table Sequences of primers used for cloning. (DOCX)

S3 Table Sequences of qRT-PCR primers. (DOCX)
