# Supplementary figures and images for: Host MOV10 is induced to restrict herpes simplex virus 1 lytic infection by promoting type I interferon response
Source: PLoS Pathog. 2022 Feb 14;18(2):e1010301. doi: 10.1371/journal.ppat.1010301 (PMC8880913; doi:10.1371/journal.ppat.1010301)

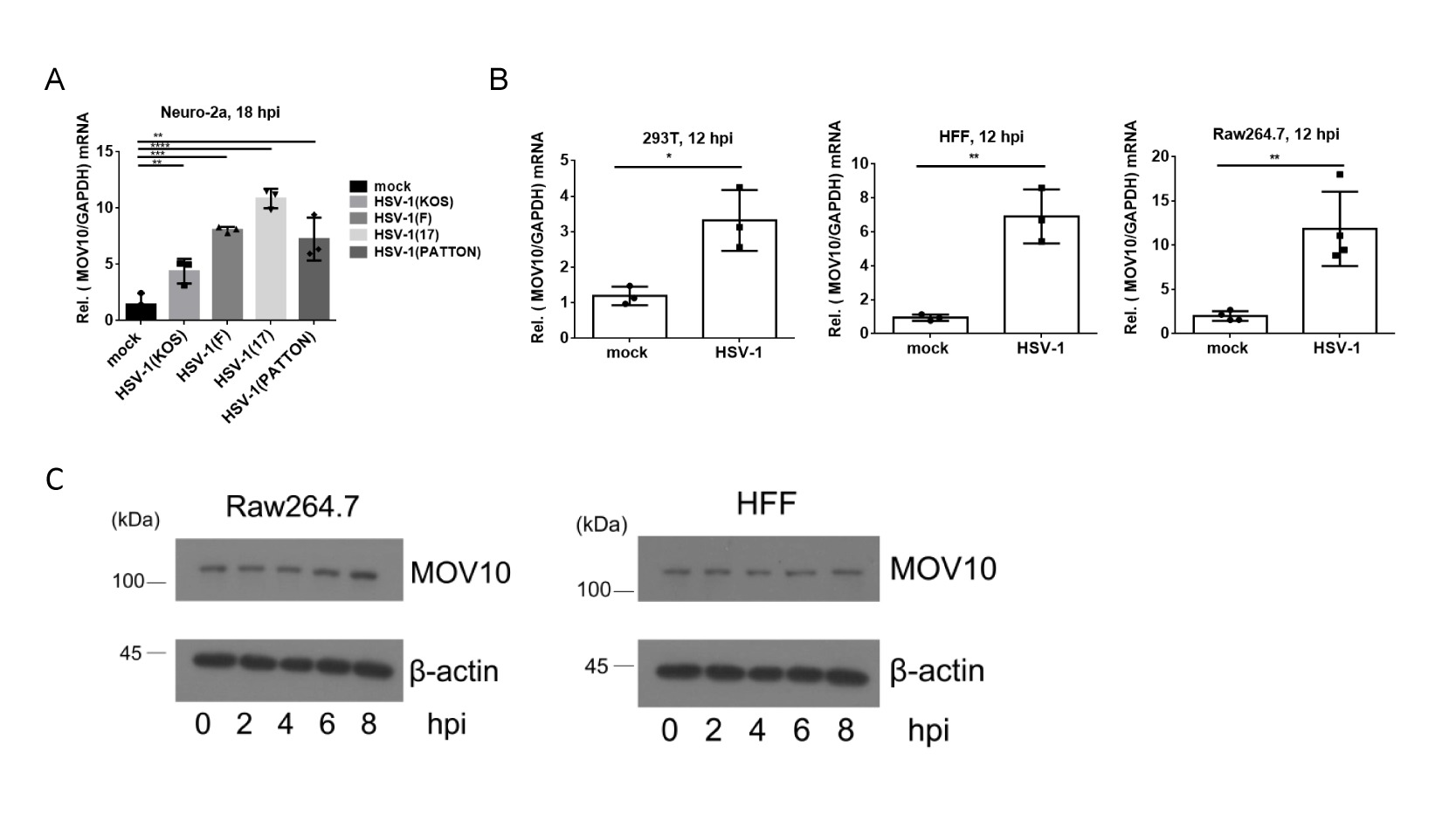

Supplement: S1 Fig — (A) Neuro-2a cells were infected with the HSV-1 strains as indicated (MOI = 5) and harvested at 18 hpi for Mov10 mRNA quantification by qRT-PCR. (B) 293T (left), HFF (middle) and Raw264.7 (right) cells were infected with HSV-1 strain KOS (MOI = 5) and harvested at 12 hpi for Mov10 mRNA quantification by qRT-PCR. (C) Raw264.7 (left) and HFF (right) cells were infected with KOS (MOI = 5) and harvested at the indicated times for MOV10 protein analysis by western blots. Data were analyzed by one-way ANOVA with Bonferroni’s multiple comparisons (A) or two-tailed unpaired t tests (B) and are presented as mean values ± SD. *, p < 0.05; **, p < 0.01; ***, p < 0.001; ****, p < 0.0001. (TIF) [file ppat.1010301.s001.tif]

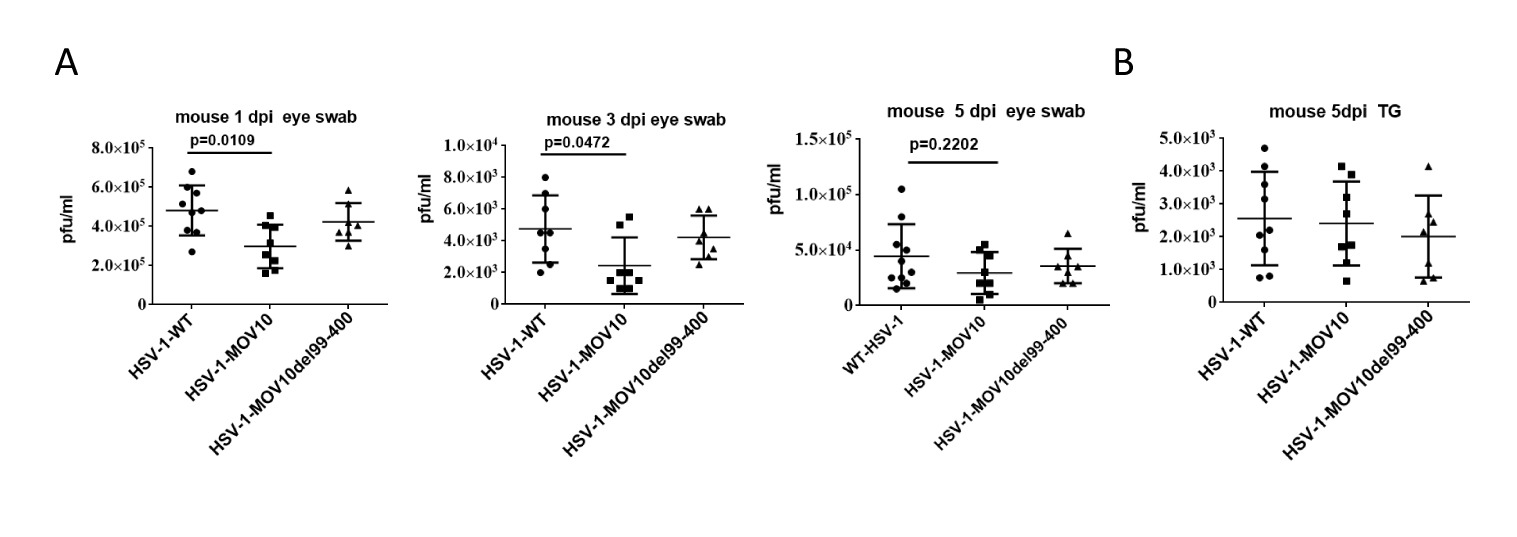

Supplement: S2 Fig — (A) Mice were inoculated on the cornea with 2 × 10^5^ pfu/eye of indicated viruses. Eye swab viral titers of WT, HSV-1-MOV10A and HSV-1-MOV10del99-400 were measured at 1 (left), 3 (middle) and 5 (right) dpi. (B) At 5 dpi, mouse TG were collected to determine titers of the indicated viruses. Data were analyzed by one-way ANOVA with Bonferroni’s multiple comparisons. Data are presented as mean values ± standard deviations (SD). (TIF) [file ppat.1010301.s002.tif]

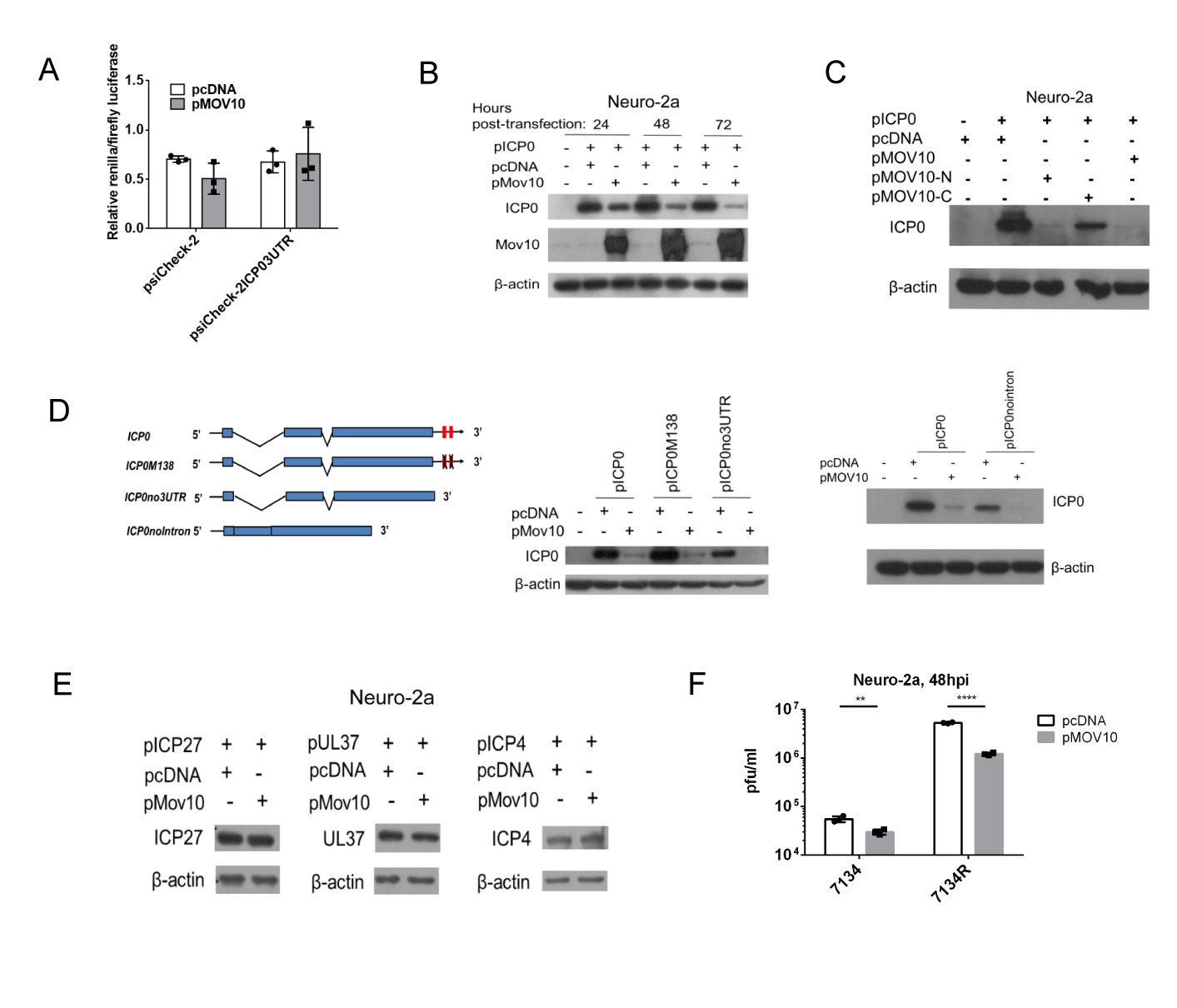

Supplement: S3 Fig — (A) Neuro-2a cells in a 24-well plate were transfected with 200 ng of pcDNA or pMOV10 and 40 ng of the empty luciferase contruct psiCheck-2 or a construct with the ICP0 3’ UTR. Luciferase was measure at 48 h post-transfection. (B) Neuro-2a cells were transfected with 150 ng of an empty vector or MOV10 expressing plasmid, together with 50 ng of an ICP0 expressing plasmid. The cells were harvested at indicated times for western blot analysis of MOV10 and ICP0. (C) Same as B except that different plasmids were used and the cells were harvested at 48 h post-transfection. (D) Left, diagram of different ICP0 expressing constructs. Blue boxes represent exons. Small red boxes represent miR-138 binding sites in the ICP0 3’ UTR. Middle and right, co-transfection was performed as in B except that different ICP0-expressing constructs as indicated at the top were used and the cells were harvested at 48 h post-transfection. (E) Same as B, but plasmids expressing different viral genes were used for co-transfection with the MOV10 expressing plasmid and the corresponding viral proteins were analyzed. (F) Neuro-2a cells were transfected with an empty vector or a MOV10 expressing plasmid for 24 h before infection with 7134 or 7134R virus (MOI = 0.1). Cells were harvested at 48 hpi for virus titration. Data were analyzed by two-way ANOVA with Bonferroni’s multiple comparisons and are presented as mean values ± SD. *, p < 0.05; **, p < 0.01; ***, p < 0.001; ****, p < 0.0001. (TIF) [file ppat.1010301.s003.tif]

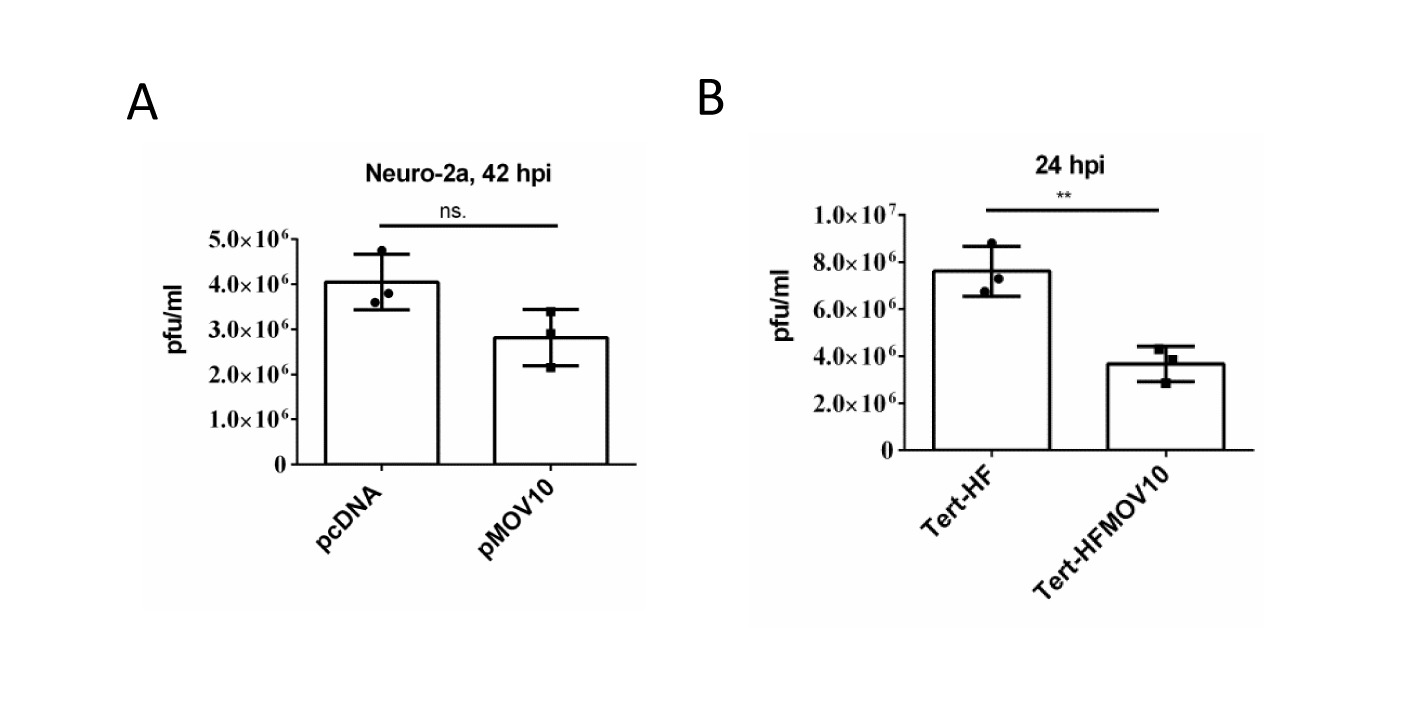

Supplement: S4 Fig — (A) Neuro-2a cells were transfected with indicated plasmid for 24 h and infected with HSV-1 (MOI = 0.5) for 12 h before collection of the supernatants. Neuro-2a cells were pretreated with these supernatants for 12 h and then infected with HSV-1 (MOI = 0.2). Viral titers were determined by plaque assays at 42 hpi. (B) Tert-HF and Tert-HFMOV10 cells were infected with HSV-1 (MOI = 0.5) for 12 h before collection of the supernatants. Neuro-2a cells were pretreated with these supernatants for 12 h and then infected with HSV-1 (MOI = 0.5). Titers were measured at 24 hpi by plaque assays. (TIF) [file ppat.1010301.s004.tif]
