# Supplementary material for: Host MOV10 is induced to restrict herpes simplex virus 1 lytic infection by promoting type I interferon response
Source: PLoS Pathog. 2022 Feb 14;18(2):e1010301. doi: 10.1371/journal.ppat.1010301 (PMC8880913; doi:10.1371/journal.ppat.1010301)
Supplement: S2 Table — (DOCX) [file ppat.1010301.s006.docx]

**S2 Table. Sequences of primers used for cloning.**

| Name | Forward primer | Reverse primer |
| --- | --- | --- |
| MOV10 | GCCTCGAGATGCCCAGTAAGTTCAGCTGC | GCGGATCCTCAGAGCTCATTCCTCCACTCTGG |
| IKKε | GCGAATTCATGCAGAGCACAGCCAATTACCT | GCGGATCCTCAGACATCAGGAGGTGCTGGGA |
| pLVX-puroMOV10 | GCGGATCCATGCCCAGTAAGTTCAGCTGCC | GCTCTAGATCAGAGCTCATTCCTCCACTCTGG |
| ICP0no3'UTR | CGACGCGTCCCCCCCTC | CCAAGCTTTTATTGTTTTCCCTCGTCCCGGG |
| ICP0nointron | CATGCCATGGAGCCCCGCC | CGACGCGTGGACTGGGG |
| MOV10Del99-200 | CATTGTAAGACCAGCTTTG | GATATCTGACCCCAGCTTC |
| MOV10Del99-300 | AGGCAGCTGCTCCCCATG | GATATCTGACCCCAGCTTCATTCTC |
| MOV10Del99-400 | TTGTCCTCGGAGACACAC | GATATCTGACCCCAGCTTC |
| MOV10Del301-400 | TTGTCCTCGGAGACACACCAG | GAGGCGGGGAGGTGGGTA |
| MOV10Del401-500 | CCAGAGCAGCTGCAGGCC | AAGGGCAAACAGGTGGTCG |
| MOV10Del498-733 | TCTCATCCCACCATCCTG | CAGACTCCGGTCGTACAG |
| MOV10Del734-939 | GAAAACGGAGGGTATACCG | CCTGTAGTTGCGGAGCAG |
| ICP27 | CGGAATTCATGGCGACTGACATTGATATGCTAA | CCAAGCTTCTAAAACAGGGAGTTGCAATAAAAA |
| ICP27-C | GACCGCATCAGCGAGAGC | GAATTCTGCAGATATCCAGCACAG |
| ICP27delRGG | AACCCGGGGGGACCCCGC | GGCAGGCTGGGCTTTGGTCGG |
