# Supplementary material for: Host MOV10 is induced to restrict herpes simplex virus 1 lytic infection by promoting type I interferon response
Source: PLoS Pathog. 2022 Feb 14;18(2):e1010301. doi: 10.1371/journal.ppat.1010301 (PMC8880913; doi:10.1371/journal.ppat.1010301)
Supplement: S3 Table — (DOCX) [file ppat.1010301.s007.docx]

**S3 Table. Sequences of qRT-PCR primers.**

| Name | qRT-PCR forward primer | qRT-PCR reverse primer |
| --- | --- | --- |
| mMOV10 | GAGGTTCGAGAGTTTTCTGGC | GCGATCTTCATTCCATACAGCAT |
| hMOV10 | TTCGAGAGTTTCCTGG | GCAATCTTCATTCCATACAGCAT |
| mGAPDH | GAAGGTCGGTGTGAACGGATT | GCCTTGACTGTGCCGTTGAA |
| hGAPDH | GAAGGTCGGAGTCAACGGATT | GCCTTGACGGTGCCATGGAA |
| ADIPSIN | TCCGGCAGCCCTCTAGT | TAGGATGACACTCGGGTATAGAC |
| mIFN-α | CTCATTCTGCAATGACCTCCACC | GACTTCTGCTCTGACCACCTCCC |
| mIFN-β | CCCTATGGAGATGACGGAGA | CTGTCTGCTGGTGGAGTTCA |
| hIFN-α | GCCTCGCCCTTTGCTTTACT | CTGTGGGTCTCAGGGAGATCA |
| hIFN-β | GCTTGGATTCCTACAAAGAAGCA | ATAGATGGTCAATGCGGCGTC |
| mISG15 | GGGTAACGATTTCCTGGTGTCCG | GAAAGGGTAAGACCGTCCTGGAGC |
| mISG54 | TACGGAAAGCAGAGGAAATCAAG | ACTGAAAGTTGCCATACCGAAGG |
| mIFNAR1 | TCGAACAAAAGACGAGGCGA | CCTTCCTCTGCTCTGACACG |
| mMDA5 | ATGGACGCAGATGTTCGTGG | TCCCTTCTCGAAGCAAGTGTC |
| mIKKε | ATGCAGAGTACCACTAACTACCT | CCTCCCCGGATTTCTTGTTTC |
| TK | ACCCGCTTAACAGCGTCAACA | CCAAAGAGGTGCGGGAGTTT |
| ICP0 | AGCGAGTACCCGCCGGCCTG | CAGGTCTCGGTCGCAGGGAAAC |
